# GSNOR plays roles in growth, pathogenicity, and stress resistance by modulating mitochondrial protein COX6B S-nitrosylation in *Colletotrichum* gloeosporioides

**DOI:** 10.1128/mbio.01269-25

**Published:** 2025-05-23

**Authors:** Xing Yang, Yongbao Yang, Aoxue Wang, Zhihao Zhao, Hongli Luo, Bang An, Qiannan Wang

**Affiliations:** 1 State Key Laboratory of Tropical Crop Breeding, School of Breeding and Multiplication (Sanya Institute of Breeding and Multiplication), School of Tropical Agriculture and Forestry (School of Agriculture and Rural Affairs & School of Rural Revitalization), Hainan University, Sanya, Hainan Province, China; Cornell University, Ithaca, New York, USA

**Keywords:** S-nitrosylation, glutathione S-nitrosoglutathione reductase (GSNOR), cytochrome c oxidase subunit 6B (COX6B), *Colletotrichum gloeosporioides*, fungal pathogenicity

## Abstract

Nitric oxide (NO) serves as a versatile signaling molecule governing diverse biological processes, primarily through post-translational modifications such as S-nitrosylation. The enzyme glutathione S-nitrosoglutathione reductase (GSNOR) plays a central role in NO homeostasis by modulating cellular levels of S-nitrosoglutathione (GSNO), thereby controlling protein S-nitrosylation dynamics. However, the functional significance of GSNOR in fungal pathogenicity remains insufficiently characterized. In this study, we investigated the function of CgGSNOR in the phytopathogenic fungus *Colletotrichum gloeosporioides*. Deletion of *CgGSNOR* disrupted nitrosative homeostasis, leading to elevated NO accumulation, increased protein S-nitrosylation levels, and mitochondrial dysfunction as evidenced by reduced ATP production and altered ROS levels. Proteomic and structural analyses identified cytochrome c oxidase subunit 6B (CgCOX6B) as a key target of S-nitrosylation. Functional characterization revealed that CgCOX6B is essential for appressorial turgor maintenance and fungal pathogenicity. Site-directed mutagenesis demonstrated that three conserved cysteine residues (Cys42, Cys62, and Cys73) are critical for CgCOX6B function and are susceptible to S-nitrosylation-induced disruption. Notably, the *CgCOX6B* knockout strain exhibited increased sensitivity to Iprodione, a widely used fungicide, and this sensitivity was further amplified by NO donor treatment. Together, our findings uncover a GSNOR-dependent redox regulatory axis that links NO signaling, mitochondrial function, and fungal pathogenicity, offering potential targets for antifungal strategies via manipulation of NO signaling networks.

**IMPORTANCE:**

*Colletotrichum gloeosporioides* is a globally significant fungal pathogen responsible for anthracnose diseases, causing losses across a wide range of crops. Although nitric oxide (NO) signaling and its post-translational regulatory mechanism, S-nitrosylation, are known to play pivotal roles in fungal biology, their specific contributions to pathogenicity remain poorly characterized. This study identifies glutathione S-nitrosoglutathione reductase (GSNOR) as a critical regulator of NO homeostasis in *C. gloeosporioides* and demonstrates its critical role in regulating fungal growth, conidiation, and pathogenicity. We uncover cytochrome c oxidase subunit 6B (COX6B) as a key target of S-nitrosylation, required for fungal energy metabolism, host infection, and resistance to fungicides. Furthermore, we reveal that exogenous NO supplementation using sodium nitroprusside synergistically enhances the antifungal activity of Iprodione. These findings advance our understanding of redox regulation in fungal pathogenesis and highlight GSNOR and COX6B as promising molecular targets for developing antifungal approaches to reduce crop losses.

## INTRODUCTION

Nitric oxide (NO) is a highly reactive and versatile signaling molecule that plays a critical role in a wide array of biological processes across diverse organisms, ranging from bacteria to humans (1, 2). The significance of NO has garnered increasing attention due to its role in modulating cellular signaling pathways, mitigating oxidative stress, and maintaining redox homeostasis. A fundamental aspect of NO’s biological function lies in its intricate interaction with reactive oxygen species (ROS) (3). Both NO and ROS are natural byproducts of cellular metabolism and engage in complex interactions that influence a wide range of signaling pathways. ROS, including superoxide anion (O_2_⁻), hydrogen peroxide (H_2_O_2_), and hydroxyl radicals (OH•), are primarily generated in mitochondria and other cellular compartments (4). While these reactive molecules act as intermediates in numerous physiological processes, their excessive accumulation can lead to oxidative damage, impairing proteins, lipids, and DNA, and ultimately disrupting cellular integrity (5).

The role of NO in plant-pathogen interactions has garnered increasing attention, as NO exhibits dual functionality, serving both defensive and pathogenic roles (6). In plants, the production of NO is induced by biotic stress factors, such as pathogen invasion, where it acts as a signaling molecule in immune responses (7). For example, NO regulates hypersensitive cell death, a hallmark of plant immunity, by activating defense-related genes and proteins (8). Moreover, NO contributes to the transcriptional regulation of resistance genes. Notably, NO induces the expression of PR-1 and WRKY family genes, which are critical components of systemic acquired resistance (SAR) in plants (9, 10). Similarly, NO signaling enhances the transcriptional activity of NPR1, a master regulator of SAR, thereby amplifying the plant’s overall immune response (11). On the other hand, pathogens, including fungi and bacteria, exploit NO signaling to enhance their pathogenicity. NO facilitates the production of virulence factors while modulating host immune responses to create a more conducive environment for infection (12, 13). In addition, NO acts as a developmental and metabolic signal to modulate asexual and sexual development, the formation of infection structures, and the production of secondary metabolites in pathogens (14, 15). The intricate balance between its defensive and pathogenic roles reflects NO’s versatility and complexity as a signaling molecule.

In living cells, NO primarily exerts its biological effects through the post-translational modification known as S-nitrosylation, a process in which NO reacts with thiol groups on cysteine residues of proteins to form S-nitrosothiols (16). S-nitrosylation is a key regulatory mechanism that modulates various aspects of protein function, including activity, stability, and molecular interactions, thereby influencing a diverse array of biological processes such as metabolism, ion transport, immune responses, and apoptosis. This modification also plays an essential role in cellular adaptation to oxidative stress (17). For example, the S-nitrosylation of antioxidant enzymes like peroxiredoxins inhibits their activity, effectively fine-tuning the cellular response to oxidative stress conditions (18).

To maintain tight regulation of NO levels and S-nitrosylation, living cells utilize the enzyme glutathione S-nitrosoglutathione reductase (GSNOR). This enzyme catalyzes the reduction of S-nitrosoglutathione (GSNO), a principal intracellular reservoir of NO, thereby controlling the extent of protein S-nitrosylation and ensuring NO homeostasis (19–21). In plants, GSNOR plays an important role in defense responses by modulating NO levels and protein S-nitrosylation during pathogen infections. For instance, in *Arabidopsis thaliana*, GSNOR regulates NO-mediated S-nitrosylation of NADPH oxidase, thereby modulating ROS production and regulating resistance plant immunity (22). Similarly, GSNOR-mediated control of NO levels influences the S-nitrosylation of salicylic acid-binding proteins NPR1, which are critical for activating systemic acquired resistance (23). Moreover, GSNOR is also crucial in mediating plant responses to various abiotic stresses such as heat, chill, drought, and salt (24–26). In animals, GSNOR has been implicated in regulating vascular tone, inflammatory responses, and oxidative stress, highlighting its critical role in maintaining overall health and homeostasis (21). Similarly, in fungi, GSNOR plays an essential role in balancing NO production and detoxification, which is vital for managing oxidative stress and ensuring fungal viability under challenging conditions (27). Moreover, in the pathogenic fungus *Magnaporthe oryzae*, GSNOR regulates appressoria formation and fungal pathogenicity by modulating the levels of nitrosylated proteins (28). These findings underscore the conserved and multifaceted role of GSNOR across diverse biological systems.

In pathogenic fungi, mitochondria play a pivotal role in facilitating pathogenicity by producing ATP to meet the energy demands required for infection and by generating ROS, which function both as signaling molecules and as agents of oxidative damage (29, 30). This dual role of mitochondria is crucial for the fungus’s ability to invade and survive within the host. For example, in *M. oryzae*, mitochondria generate ROS during the formation of appressoria, which is important for cell development (31). Similarly, in *Cryptococcus neoformans*, mitochondrial dynamics and ATP production have been shown to directly influence fungal pathogenicity and its survival within the host (32, 33). In *Botrytis cinerea*, mitochondria-generated ROS are required for polarity growth and pathogenicity (34). In our previous work, ROS was also found to be required for the formation of appressoria in *Colletotrichum gloeosporioides* (35, 36).

Recently, S-nitrosylation of mitochondrial proteins has emerged as a critical regulatory mechanism in maintaining mitochondrial function. This post-translational modification influences key processes such as ATP production, ROS management, and apoptosis (37). In animal models, S-nitrosylation of electron transport chain (ETC) proteins is involved in mitochondrial respiration and ROS production (38). For example, S-nitrosylation of Complex I subunits in mitochondria could inhibit mitochondrial functions (39, 40). These observations suggest that S-nitrosylation of mitochondrial proteins might be a key factor in fungal pathogenicity by fine-tuning mitochondrial activity during infection. Consequently, targeting S-nitrosylation pathways and mitochondrial dynamics might present a promising avenue for developing novel strategies to control fungal diseases.

*C. gloeosporioides* is a significant fungal pathogen responsible for anthracnose diseases affecting a wide variety of crops. Its infection process begins with the formation of appressoria and subsequent tissue invasion, both of which are regulated by signaling pathways involving ROS (35, 36). While significant progress has been made in understanding the roles of NO and S-nitrosylation as critical regulators of cellular signaling, the precise role of GSNOR in *C. gloeosporioides* remains insufficiently explored. In addition, the regulatory functions of mitochondrial S-nitrosylation in fungal pathogenicity are largely uncharted, and the identification of mitochondrial proteins targeted by S-nitrosylation in phytopathogenic fungi remains unexplored. Addressing these knowledge gaps is vital for unraveling the molecular mechanisms governing fungal pathogenicity and could provide a foundation for developing targeted approaches to mitigate anthracnose diseases more effectively.

In this study, we explored the role of CgGSNOR in regulating NO signaling and its impact on the pathogenicity of *C. gloeosporioides*. Through comprehensive proteomic analysis, we identify the mitochondrial protein CgCOX6B as a key target of S-nitrosylation. Furthermore, our findings reveal that CgCOX6B is essential for the pathogen’s pathogenicity and fungicide resistance. These results highlight the significance of NO-mediated post-translational modifications in regulating mitochondrial function and fungal pathogenicity, offering potential avenues for targeted disease management strategies.

## RESULTS

### Identification of CgGSNOR and construction of the mutant strains

A BLAST search against the *C. gloeosporioides* genome identified a gene homologous to the *GSNOR* genes from *Saccharomyces cerevisiae*, which was designated as *CgGSNOR*. The predicted *CgGSNOR* gene contains an open reading frame (ORF) of 1,146 bp, encoding a protein of 381 amino acids. Structural domain analysis revealed the presence of two highly conserved domains characteristic of GSNOR family proteins: the alcohol dehydrogenase GroES-like domain (ADH_N) and the zinc-binding dehydrogenase domain (ADH_zinc_N) (Fig. S1a). Phylogenetic analysis further demonstrated that *CgGSNOR* is highly conserved across fungal species, particularly those pathogenic to plants. The *CgGSNOR* gene clusters closely with homologs from well-known plant-pathogenic fungi such as *Fusarium graminearum* and *Verticillium dahliae* (Fig. S1b). To further validate the evolutionary conservation of *CgGSNOR*, a multiple sequence alignment was performed. The alignment revealed a high degree of conservation in critical residues within both the ADH_N and ADH_zinc_N domains, underscoring the functional importance of these regions in maintaining the enzyme’s activity (Fig. S1C).

To investigate the biological roles of *CgGSNOR* in *C. gloeosporioides*, we employed the split-marker recombination strategy to create a targeted knockout of the *CgGSNOR* gene. The resulting mutant strain was designated Δ*CgGSNOR*. To confirm the mutant phenotype, we performed a complementation experiment in which the *CgGSNOR* gene, driven by its native promoter, was reintroduced into the mutant strain. The complemented strain was designated Res-Δ*CgGSNOR* (Fig. S2).

### Functional analysis of *CgGSNOR* in colony morphology, conidiation, and germination

**Fig 1:**
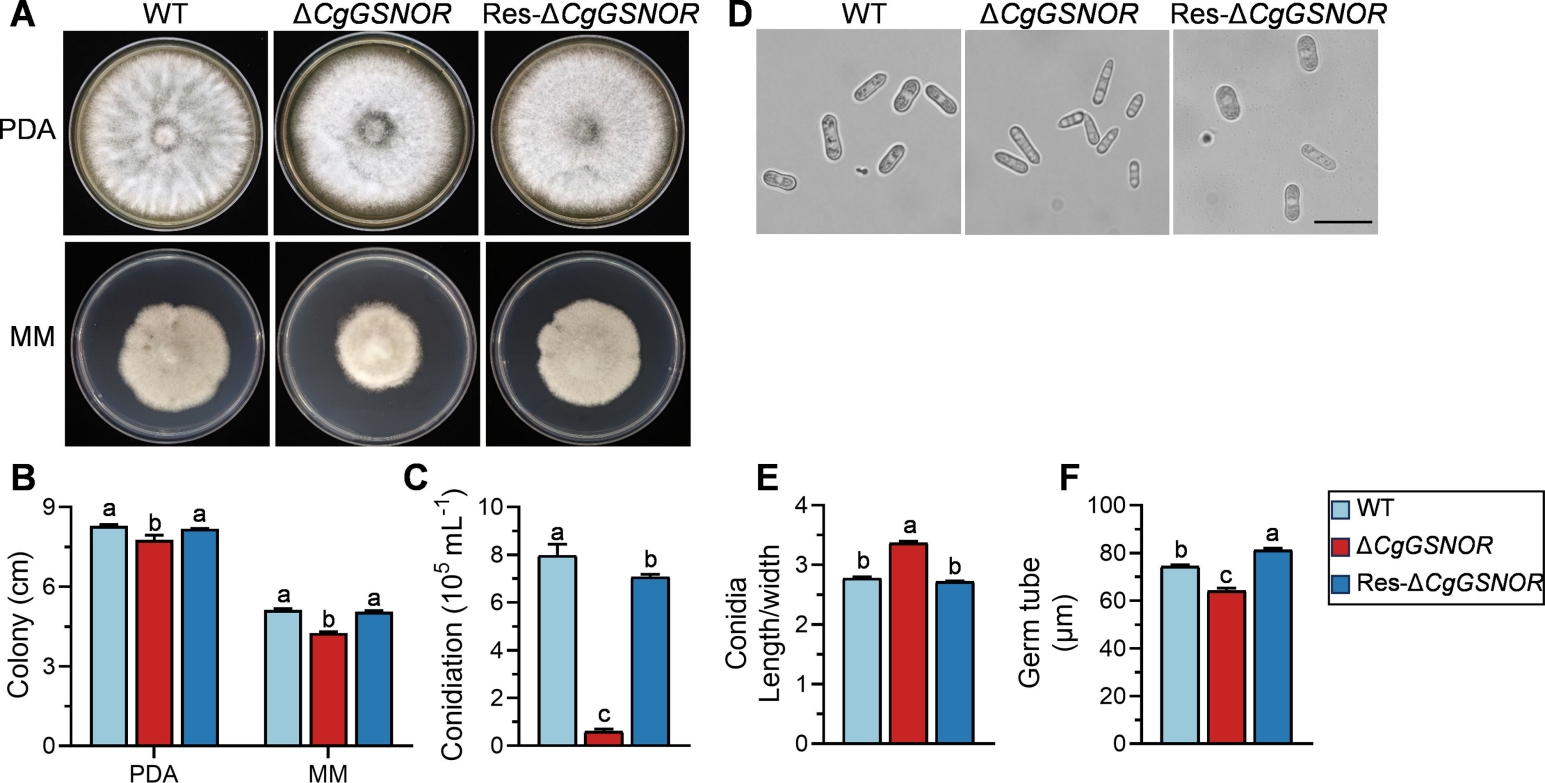
Phenotypic analyses of the *CgGSNOR* mutant strains. (**A**) Colony morphology of wild-type (WT), Δ*CgGSNOR*, and Res-Δ*CgGSNOR* strains grown on PDA and MM plates after 5 d of incubation. (**B**) Colony diameter of WT, Δ*CgGSNOR*, and Res-Δ*CgGSNOR* strains on PDA and MM plates. (**C**) Conidiation of WT, Δ*CgGSNOR*, and Res-Δ*CgGSNOR* in liquid complete medium. (**D**) Microscopic images of conidia from WT, Δ*CgGSNOR*, and Res-Δ*CgGSNOR*. Scale bar = 20 µm. (**E**) Conidia length-to-width ratio for WT, Δ*CgGSNOR*, and Res-Δ*CgGSNOR*. (**F**) Germ tube length of WT, Δ*CgGSNOR*, and Res-Δ*CgGSNOR* strains after incubation for 4 h. Bars represent the mean ± standard deviation from three independent replicates. Different letters above bars indicate statistically significant differences (*P* < 0.05).

The colony morphology and growth rates of the WT, Δ*CgGSNOR*, and Res-Δ*CgGSNOR* strains were analyzed on potato dextrose agar (PDA) and minimal medium (MM) plates (Fig. 1A). The Δ*CgGSNOR* mutant exhibited significantly reduced colony diameters on both PDA and MM compared to the WT strain (Fig. 1B). In addition, the Δ*CgGSNOR* mutant produced significantly fewer conidia, with a more than sevenfold reduction in conidial concentration compared to the WT and Res-Δ*CgGSNOR* strains (Fig. 1C). Furthermore, we observed alterations in conidial morphology in the Δ*CgGSNOR* mutant. The conidia of the Δ*CgGSNOR* mutant exhibited an increased length-to-width ratio compared to the WT and Res-Δ*CgGSNOR* strains (Fig. 1D and E), indicating a morphological change in the conidia. To investigate the impact of *CgGSNOR* deletion on conidial germination, we measured germ tube length and found that the Δ*CgGSNOR* mutant exhibited significantly shorter germ tubes compared to the WT and Res-Δ*CgGSNOR* strains (Fig. 1F). This reduced germ tube length indicates a defect in early germination processes, although the germination rate itself was not impaired.

### Pathogenicity and infection structure development of *CgGSNOR* mutant strains

**Fig 2:**
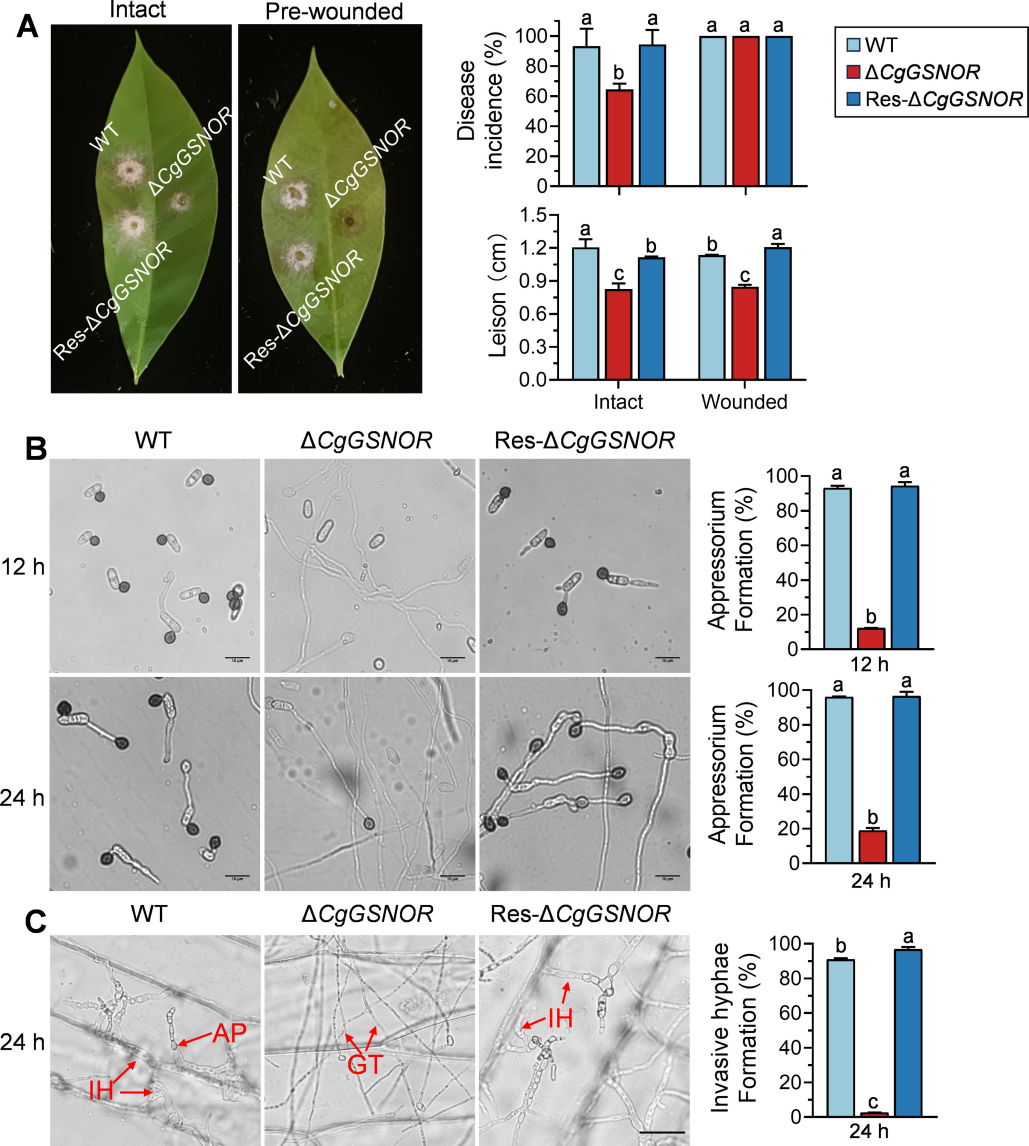
Pathogenicity assay and appressorium formation of WT, Δ*CgGSNOR*, and Res-Δ*CgGSNOR* strains. (**A**) Pathogenicity assessment of the WT, Δ*CgGSNOR*, and Res-Δ*CgGSNOR* strains on intact and pre-wounded rubber tree leaves. The disease symptoms were recorded at 3 d post-inoculation. The bar charts show the disease incidence and lesion diameters. (**B**) Appressorium formation of the mutant strains on artificial hydrophobic surfaces at 12 and 24 h post-inoculation. Scale bars = 20 µm. The bar chart shows the appressorium formation percentages. (**C**) Penetration assay on onion epidermis at 24 h post-inoculation. Germ tube (GT) and invasive hyphae (IH) are marked with arrows. Scale bars = 20 µm. The bar chart shows the IH formation percentage. Bars represent the mean ± standard deviation from three independent replicates. Different letters above bars indicate statistically significant differences (*P* < 0.05).

The pathogenicity of the WT, Δ*CgGSNOR*, and Res-Δ*CgGSNOR* strains was assessed on both intact and pre-wounded rubber tree leaves (Fig. 2A). On intact leaves, the Δ*CgGSNOR* mutant exhibited significantly lower disease incidence and smaller lesion sizes than the WT and Res-Δ*CgGSNOR* strains. Meanwhile, on pre-wounded leaves, the Δ*CgGSNOR* mutant showed a reduced disease incidence compared to the other strains, and it produced significantly smaller lesions compared to the WT and Res-Δ*CgGSNOR* strains. This result suggests that while the initial infection process might be unaffected, the mutant strain is impaired in lesion development. By contrast, on intact leaves, the Δ*CgGSNOR* mutant exhibited significantly lower disease incidence and smaller lesion sizes than the WT and Res-Δ*CgGSNOR* strains, indicating that *CgGSNOR* is crucial for efficient infection and lesion progression in non-wounded tissues.

To investigate whether the infection process was impaired in the Δ*CgGSNOR* mutant, we examined appressorium formation by incubating conidia from *C. gloeosporioides* on hydrophobic polyester surfaces (Fig. 2B). After 12 h of incubation, approximately 90% of the conidia from the WT and Res-Δ*CgGSNOR* strains formed short germ tubes that differentiated into dome-shaped appressoria, while only 10% of the conidia from the Δ*CgGSNOR* mutant formed appressoria. The appressoria formed by the mutant exhibited abnormal morphology with long, poorly developed germ tubes. After 24 h, appressorium formation remained significantly reduced in the Δ*CgGSNOR* mutant, and the appressoria that formed were still abnormal. These results indicate that *CgGSNOR* is essential for normal appressorium formation, a critical process for fungal penetration of host tissues. Next, we performed a penetration assay on onion epidermis to assess the ability of the Δ*CgGSNOR* mutant to penetrate the host tissue. After 24 h of incubation, over 80% of the conidia from the WT and Res-Δ*CgGSNOR* strains successfully formed appressoria that developed into invasive hyphae (IH). By contrast, the Δ*CgGSNOR* mutant produced long germ tubes that grew on the onion surface but failed to penetrate, with less than 5% of the conidia forming invasive hyphae (Fig. 2C).

### CgGSNOR regulates NO accumulation, O_2_⁻ levels, and ATP production

**Fig 3:**
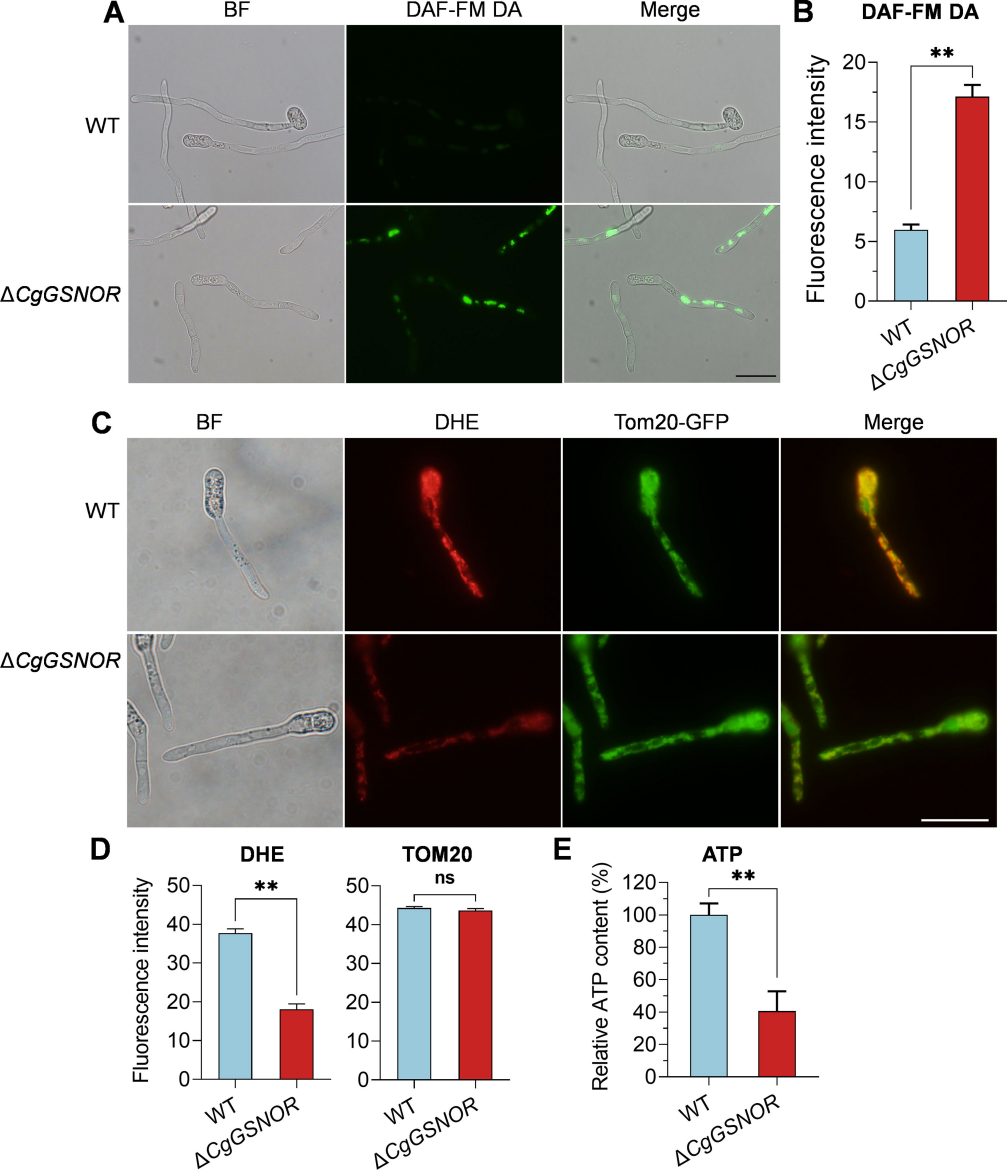
Detection of nitric oxide (NO) accumulation, superoxide (O_2_⁻) levels, and ATP production. (**A**) Detection of NO levels in WT and Δ*CgGSNOR* strains using the NO-sensitive fluorescent probe DAF-FM DA. BF: bright field. Scale bars = 20 µm. (**B**) Quantification of DAF-FM DA fluorescence intensity. (**C**) Detection of O_2_⁻ using DHE staining and co-localization with mitochondria using Tom20-GFP expression strains. (**D**) Quantification of DHE and TOM20-GFP fluorescence intensities. (**E**) Relative ATP content in WT and Δ*CgGSNOR* strains measured using a luciferase-based ATP assay kit. Relative fluorescence intensity was calculated with ImageJ. Bars represent the mean ± standard deviation from three independent samples, and each sample contains at least 20 hyphae. Asterisks indicate significant differences (*P < 0.05, **P < 0.01; ns, not significant).

To evaluate the role of CgGSNOR in redox homeostasis, we first assessed NO levels in *C. gloeosporioides* using the fluorescent probe DAF-FM DA. Microscopic analysis revealed a markedly stronger fluorescence signal in the Δ*CgGSNOR* mutant compared to the WT strain, indicating elevated NO accumulation in the Δ*CgGSNOR* mutant (Fig. 3A and B). Next, we examined O_2_⁻ levels using dihydroethidium (DHE) staining. In WT hyphae, DHE fluorescence co-localized with the mitochondrial outer membrane marker TOM20-GFP (Fig. S3), whereas the Δ*CgGSNOR* mutant exhibited a significant reduction in DHE fluorescence despite similar TOM20-GFP signal intensity (Fig. 3C and D). This suggests that while mitochondrial abundance or structure was not noticeably altered by the loss of CgGSNOR, mitochondrial ROS production was impaired. Consistent with this, we found that ATP levels were significantly reduced in the Δ*CgGSNOR* mutant compared to the WT strain (Fig. 3E), indicating a defect in mitochondrial energy metabolism.

### CgGSNOR regulates NO-dependent appressorium formation, turgor maintenance, and protein S-nitrosylation

**Fig 4:**
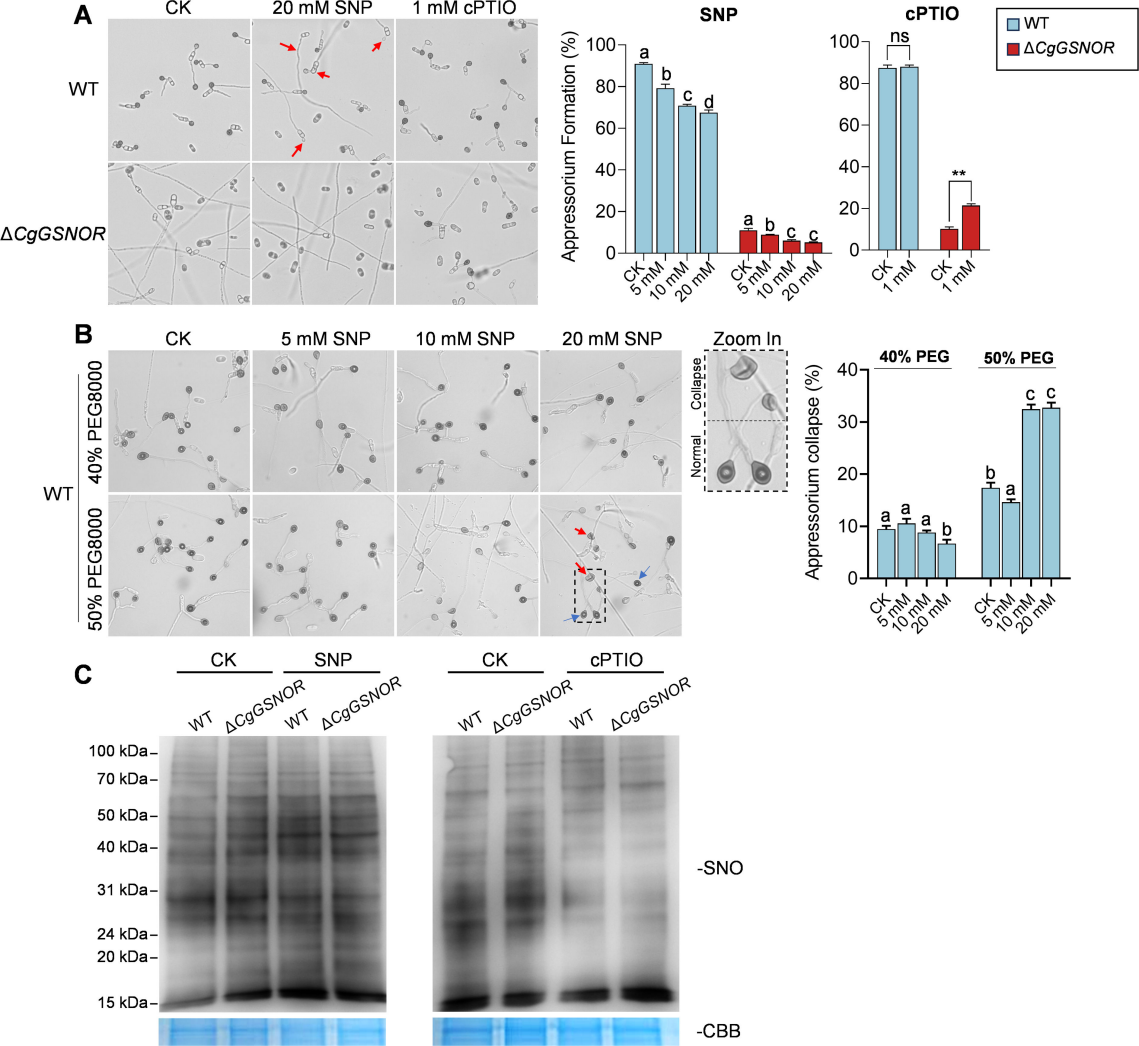
Analysis of nitric oxide (NO) effect on appressorium development, turgor maintenance, and protein S-nitrosylation. (**A**) Appressorium formation assay in WT and Δ*CgGSNOR* strains treated with the NO donor sodium nitroprusside (SNP) or the NO scavenger Carboxy-PTIO (cPTIO). Red arrows indicate abnormal appressoria. The bar chart shows the appressorium formation percentages. (**B**) Turgor pressure maintenance of WT appressoria in the presence of 40% and 50% PEG8000, with or without SNP treatment. The normal and collapsed appressoria are indicated with blue and red arrows, respectively. Zoomed-in images show representative examples of normal versus collapsed appressoria. The bar chart shows the percentage of collapsed appressoria. Bars represent the mean ± standard deviation from three independent samples. Different letters indicate statistically significant differences (*P* < 0.05), and asterisks indicate significant differences (*P < 0.05, **P < 0.01; ns, not significant). (**C**) Western blot analysis of protein S-nitrosylation (SNO) in WT and Δ*CgGSNOR* strains treated with SNP or cPTIO. Protein loading was confirmed using Coomassie Brilliant Blue (CBB) staining.

To investigate whether NO accumulation contributes to the impaired appressorium development in the Δ*CgGSNOR* mutant, we treated conidia with an NO donor, sodium nitroprusside (SNP), and an NO scavenger, Carboxy-PTIO (cPTIO). In WT conidia, SNP treatment significantly reduced appressorium formation in a concentration-dependent manner, whereas treatment with cPTIO did not significantly affect appressorium formation. By contrast, Δ*CgGSNOR* showed consistently low appressorium formation across treatments. Notably, cPTIO treatment partially rescued the appressorium formation defect in the Δ*CgGSNOR* mutant (Fig. 4A). To further explore the functional consequences of NO on appressorium turgor, we performed an appressorium collapse assay in the presence of 40% and 50% polyethylene glycol (PEG8000). The WT strain maintained appressorium integrity under native conditions, while SNP treatment significantly increased the proportion of collapsed appressoria in a dose-dependent manner (Fig. 4B). These results suggest that NO accumulation negatively affects appressorium development and appressorium turgor integrity. We also evaluated global protein S-nitrosylation levels under SNP and cPTIO treatments. Western blot analysis revealed that S-nitrosylation was elevated in Δ*CgGSNOR* compared to WT under native conditions. In addition, SNP treatment increased global S-nitrosylation in both strains, with a more pronounced effect in Δ*CgGSNOR*. Conversely, cPTIO treatment reduced S-nitrosylation levels (Fig. 4C), confirming the NO-dependence of S-nitrosylation modification.

### Analysis of mitochondrial protein S-nitrosylation and proteomic profiling of S-nitrosylated proteins

Mitochondria were isolated from both WT and Δ*CgGSNOR* strains following the manufacturer’s protocols for mitochondrial enrichment. The purity of the isolated mitochondria was confirmed through SDS-PAGE analysis, which demonstrated clear separation between the mitochondrial and cytoplasmic proteins. In addition, Western blotting using specific mitochondrial and cytoplasmic markers validated the high quality of the mitochondrial preparations (Fig. S4).

**Fig 5:**
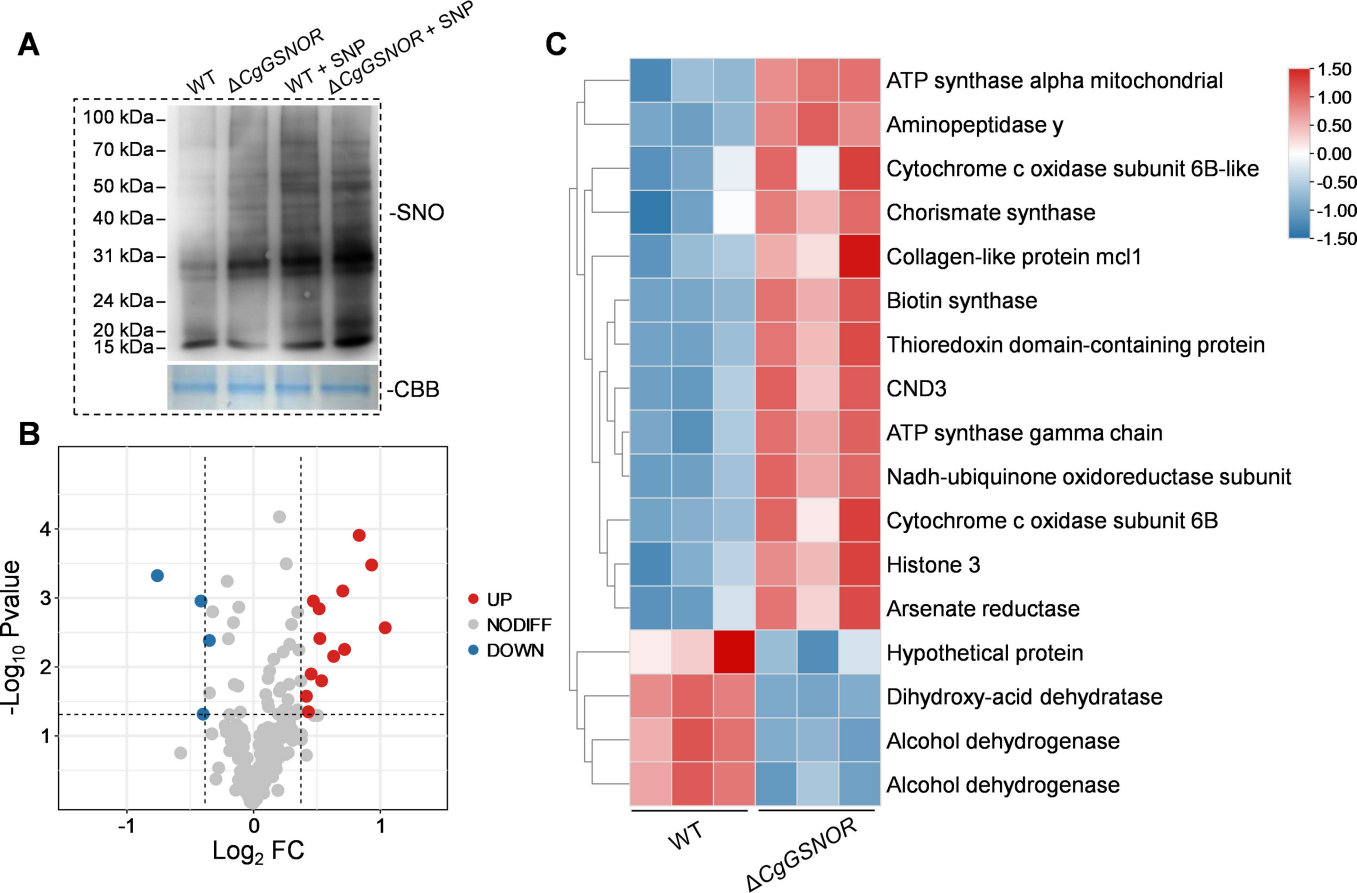
Proteomic analysis of S-nitrosylated mitochondrial proteins in *C. gloeosporioides* strains. (**A**) Western blot analysis of mitochondrial protein S-nitrosylation (SNO) in WT and Δ*CgGSNOR* strains under untreated and sodium nitroprusside (SNP)-treated conditions. Protein loading was confirmed using Coomassie Brilliant Blue (CBB) staining. (**B**) Volcano plot of the proteomic analysis showing the differential regulation of S-nitrosylated proteins between Δ*CgGSNOR* and WT strains. (**C**) Heatmap showing the relative level of S-nitrosylation of the different regulated mitochondrial proteins.

To evaluate the impact of CgGSNOR on mitochondrial protein S-nitrosylation, we examined the levels of protein S-nitrosylation under both native and NO-inducing conditions. As shown in Fig. 5A, the Δ*CgGSNOR* mutant exhibited markedly higher levels of S-nitrosylated proteins than the WT strain under native conditions. Treatment with SNP further increased S-nitrosylation levels in both strains, with a more pronounced effect observed in Δ*CgGSNOR*. To identify specific targets of S-nitrosylation, we conducted a comprehensive proteomic analysis of the mitochondrial fractions. A total of 207 distinct S-nitrosylated cysteine residues, corresponding to 155 different proteins, were identified (Table S1). Among these, 4 cysteine residues from 3 proteins exhibited reduced S-nitrosylation levels in the Δ*CgGSNOR* mutant, whereas 13 proteins showed significantly increased S-nitrosylation in the mutant compared to the WT (Fig. 5B and (Table S2). Notably, the upregulated proteins included key components of the ETC and several enzymes involved in secondary metabolite synthesis. The heatmap illustrates a broad shift in mitochondrial S-nitrosylation associated with *CgGSNOR* deletion, particularly affecting proteins essential for energy metabolism and redox balance (Fig. 5C).

### Functional validation of Cytochrome c oxidase subunit 6B (COX6B)

Then, we set out to validate the roles of key components of the ETC identified in the proteomic analysis. Three proteins were selected for further examination: Cytochrome c oxidase subunit 6B (CgCOX6B), Cytochrome c oxidase subunit 6B-like (CgCOX6BL), and a Thioredoxin domain-containing protein (CgTRX) (Table S1). In addition, since the ATP synthase alpha and gamma subunits (ATP5F1A and ATP5F1C) are well known to be essential in various species, we also selected the ATP synthase alpha subunit for subsequent validation. After constructing the respective knockout mutants, we performed colony growth assays. The results showed that the knockout of *CgCOX6B* (Fig. S5) led to a significant reduction in colony growth, while the mutants for *CgCOX6BL* and *CgTRX* did not exhibit significant differences in growth compared to the WT strain. Notably, we were unable to generate a mutant for the ATP synthase alpha subunit, suggesting that it is essential for the survival and growth of *C. gloeosporioides* (Fig. S6). As a result, CgCOX6B was selected for further analysis.

**Fig 6:**
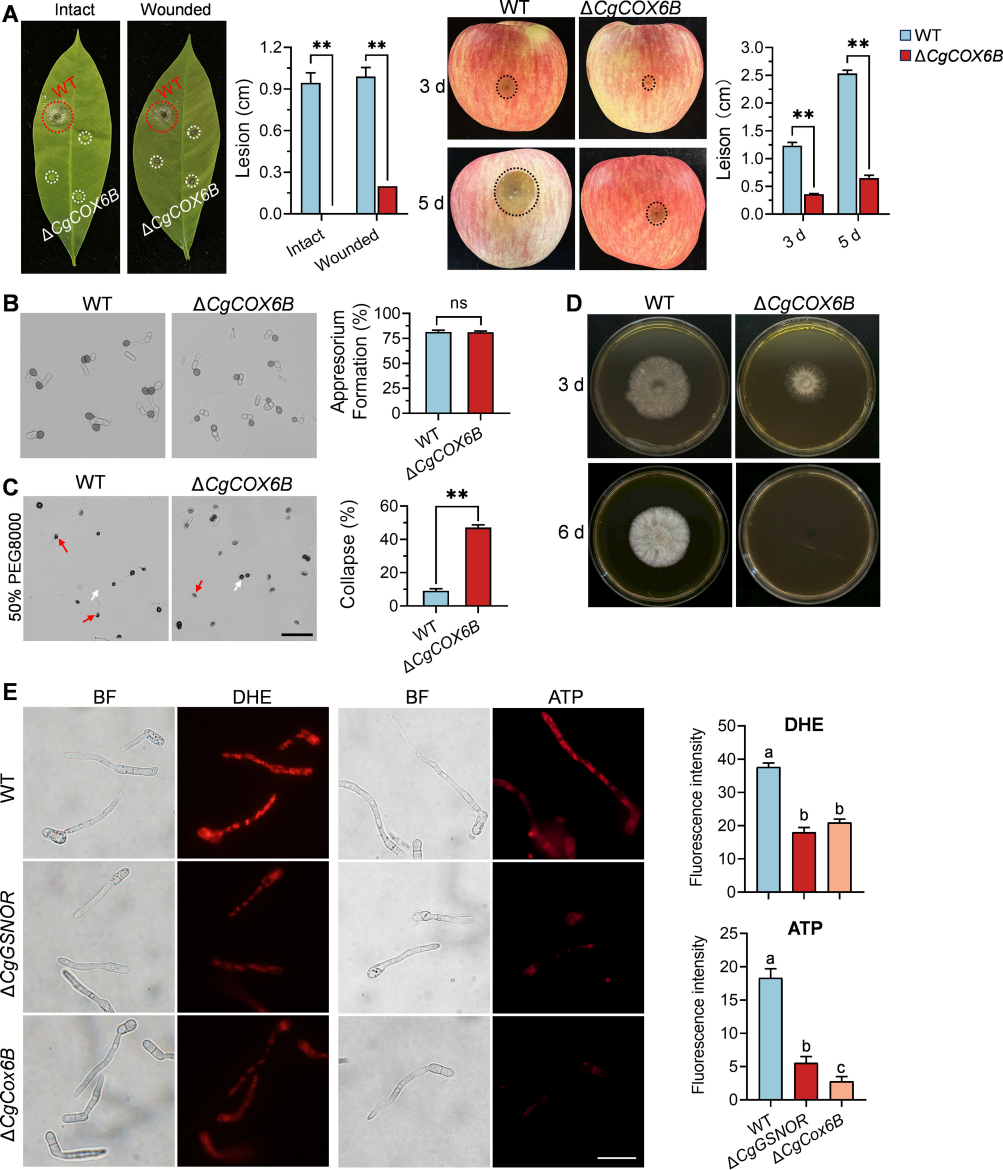
Pathogenicity, appressorium formation, and penetration ability of the Δ*CgCOX6B* mutant. (**A**) Pathogenicity assays on intact and wounded rubber tree leaves and apple fruit inoculated with the WT and Δ*CgCOX6B* strains. The bar charts show the lesion diameters on leaves at 3 d post-inoculation and lesion diameters on apple fruits at 3 and 5 d post-inoculation. (**B**) Appressorium formation of the WT and Δ*CgCOX6B* strains on polyester surfaces at 12 h post-inoculation. The bar chart shows the appressorium formation percentages. (**C**) Turgor pressure maintenance of appressoria in the presence of 50% PEG8000. The normal and collapsed appressoria are indicated with white and red arrows, respectively. The bar chart shows the percentage of collapsed appressoria under treatment with 50% PEG8000. (**D**) Penetration assay on cellophane. After 3 d of incubation, the colonies along with the cellophane were removed from the PDA plates, and the plates were incubated for another 3 d. (**E**) Mitochondrial superoxide (O2⁻) levels and ATP content were assessed in WT, Δ*CgGSNOR*, and Δ*CgCOX6B* strains. Superoxide levels were measured using DHE staining, and ATP content was visualized by a fluorescent ATP probe ATP-RED 1. Scale bars = 20 µm. Bars represent the mean ± standard deviation from three independent samples. Different letters indicate statistically significant differences (*P* < 0.05), and asterisks indicate significant differences (*P < 0.05, **P < 0.01; ns, not significant).

To further investigate the role of *CgCOX6B* in fungal pathogenesis, we conducted pathogenicity assays on both intact and wounded rubber tree leaves and apple fruit. The results demonstrated that the Δ*CgCOX6B* mutant produced dramatically smaller lesions on wounded leaves compared to the WT strain. Furthermore, the mutant completely lost its ability to infect intact leaves (Fig. 6A). On apple fruit, the Δ*CgCOX6B* mutant produced considerably smaller lesions, with a dramatic reduction in lesion size observed at both 3 and 5 d post-inoculation (Fig. 6A). Next, we evaluated the role of CgCOX6B in infection structure development. The results showed that the Δ*CgCOX6B* mutant exhibited no significant differences in appressorium formation percentage compared to the WT strain (Fig. 6B). We next examined the ability of the appressoria to maintain turgor pressure under osmotic stress. In the presence of 50% PEG8000, the Δ*CgCOX6B* mutant displayed a significantly higher proportion of collapsed appressoria than the WT strain, indicating a reduced ability of the Δ*CgCOX6B* mutant to maintain turgor pressure during infection-related processes (Fig. 6C). To assess invasive capacity, we performed a cellophane penetration assay. After incubating the strains on cellophane for 3 days, the colony, along with the cellophane, was removed from the PDA plates. Subsequent examination revealed that the WT strain successfully penetrated the cellophane, while the Δ*CgCOX6B* mutant failed to do so, indicating a severe defect in substrate invasion (Fig. 6D). To explore the underlying cause of these defects in the Δ*CgCOX6B* mutant, we examined mitochondrial function in the Δ*CgCOX6B* mutant. DHE staining revealed a significant reduction in mitochondrial ROS generation compared to the WT. In addition, ATP staining showed that the Δ*CgCOX6B* mutant had markedly reduced ATP content compared to WT, and lower than that of Δ*CgGSNOR*, indicating that CgCOX6B is essential for maintaining mitochondrial energy output and redox homeostasis (Fig. 6E).

### Structural modeling reveals conformational changes in CgCOX6B induced by S-nitrosylation

**Fig 7:**
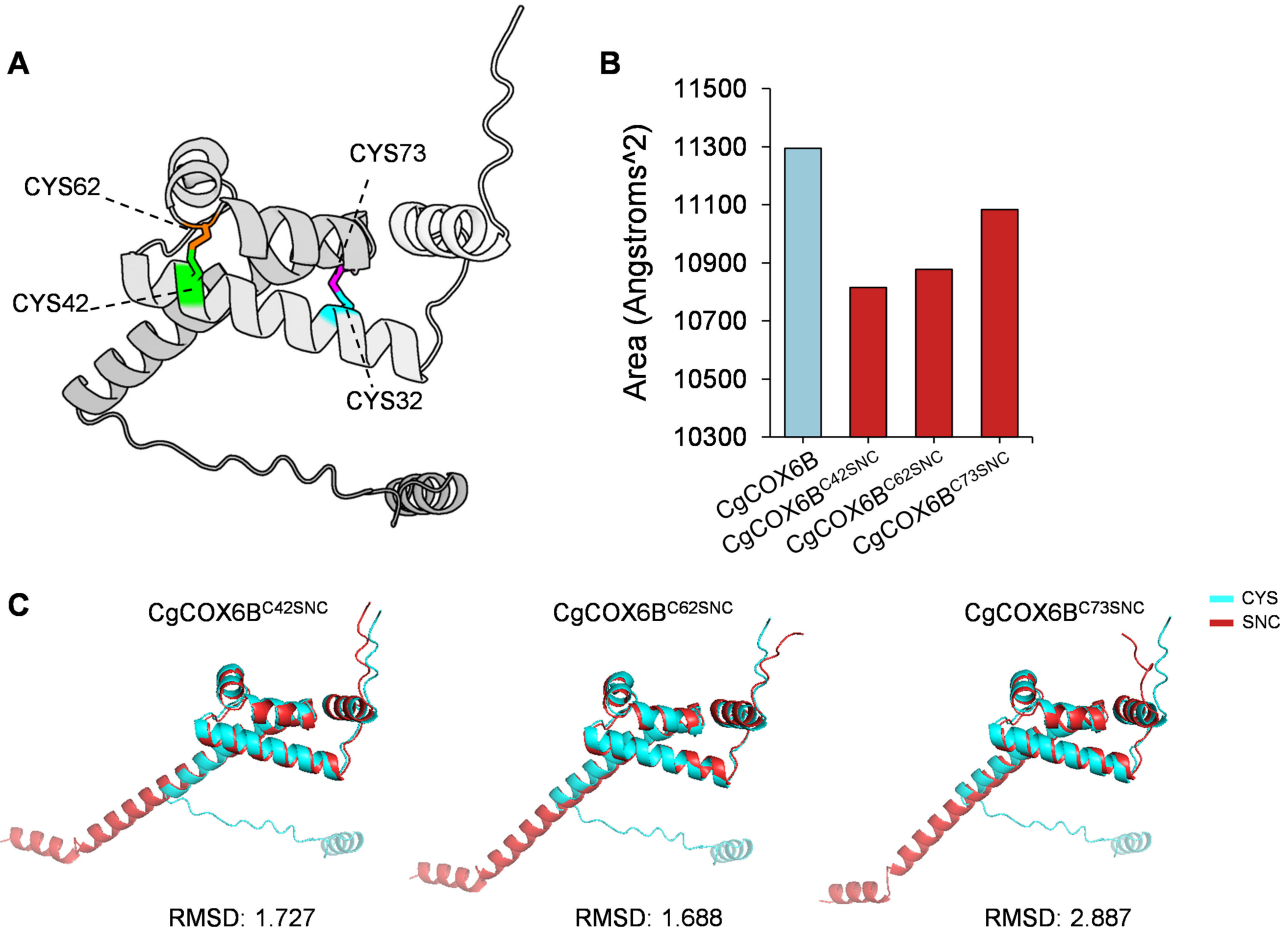
Structural modeling and conformational impact of S-nitrosylation on key cysteine residues in CgCOX6B. (**A**) Predicted 3D structure of CgCOX6B showing the positions of four conserved cysteine residues: Cys32, Cys42, Cys62, and Cys73, highlighted in different colors. (**B**) Solvent-accessible surface area (SASA) of wild-type CgCOX6B and three S-nitrosylated variants (C42SNC, C62SNC, and C73SNC). (**C**) Structural alignment of wild-type and three S-nitrosylated variants of CgCOX6B. Root mean square deviation (RMSD) values are shown for each variant.

Proteomic data indicated that *CgCOX6B* harbors S-nitrosylation modifications at the Cys42, Cys62, and Cys73 residues, with Cys73 exhibiting an upregulated modification level in the Δ*CgGSNOR* mutant compared to WT (Table S1). These findings raised the possibility that these residues contribute to the functional role of CgCOX6B. To investigate the structural consequences of S-nitrosylation on CgCOX6B, we performed comparative structural analysis of wild-type and S-nitrosylated variants at the three key cysteine residues. Structural prediction revealed that Cys42–Cys62 and Cys32–Cys73 form two disulfide bridges that likely contribute to the stabilization of the helical bundle architecture in CgCOX6B (Fig. 7A). S-nitrosylation of the cysteine residues at the modification sites of target proteins resulted in S-nitroso-cysteine (SNC). We first analyzed the solvent-accessible surface area (SASA) of wild-type CgCOX6B and its S-nitrosylated forms. A notable reduction in SASA was observed upon S-nitrosylation at each site, particularly in the CgCOX6B^C42SNC^ and CgCOX6B^C62SNC^ variants (Fig. 7B), suggesting that S-nitrosylation at disulfide-forming cysteines induces structural compaction and alters solvent exposure. To further evaluate conformational deviations, we calculated the root mean square deviation (RMSD) between the wild-type and each S-nitrosylated model. The RMSD values were 1.727 for CgCOX6B^C42SNC^, 1.688 for CgCOX6B^C62SNC^, and 2.887 for CgCOX6B^C73SNC^ (Fig. 7C), indicating significant structural alterations. These findings imply that S-nitrosylation at specific cysteine residues alters the structural integrity of CgCOX6B.

### Functional analysis of cysteine residues in CgCOX6B

**Fig 8:**
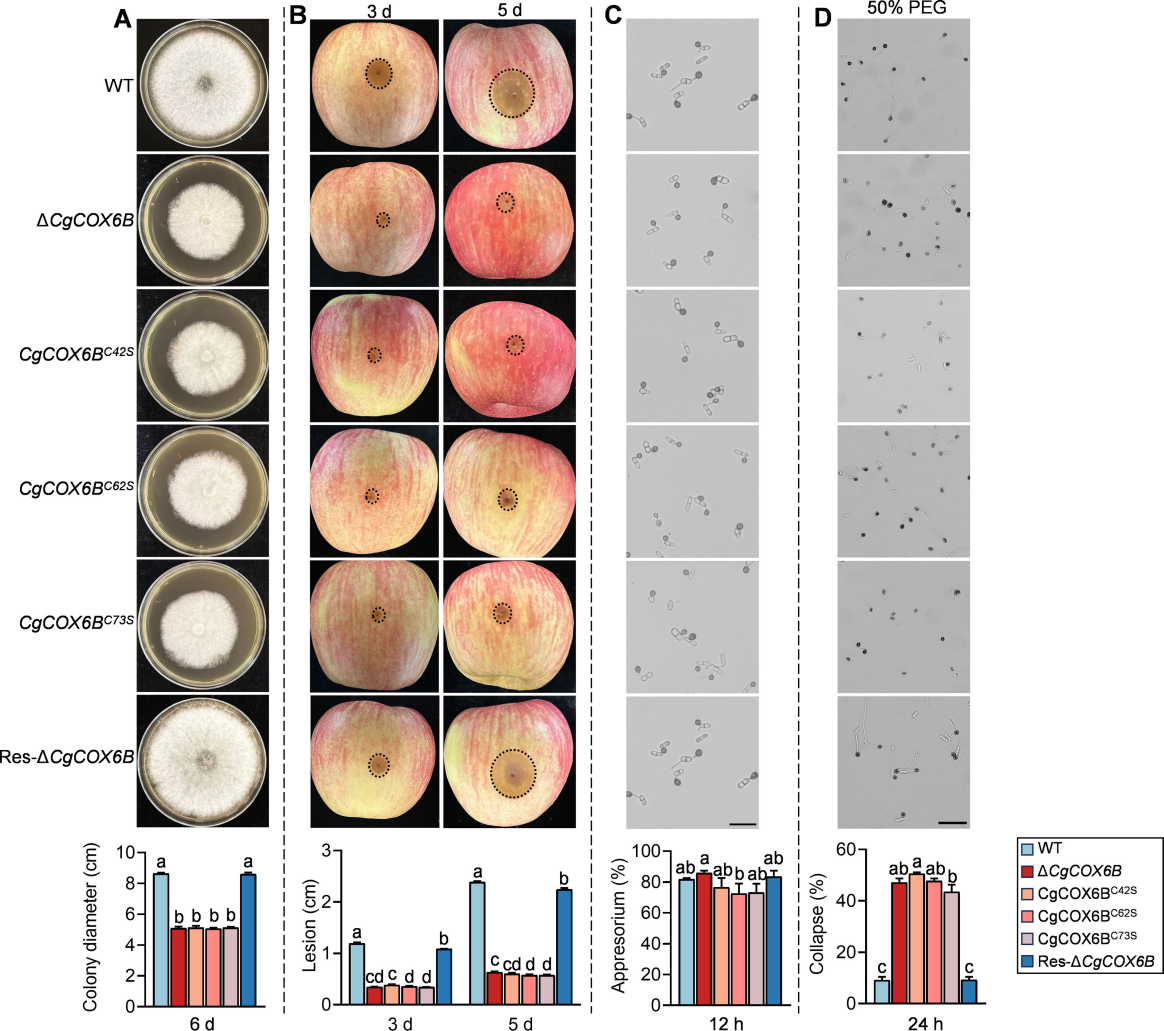
Functional analysis of cysteine residues in CgCOX6B. (**A**) Colony growth of the WT, Δ*CgCOX6B*, and cysteine site mutants, CgCOX6B^C42S^, CgCOX6B^C62S^, and CgCOX6B^C73S^, on PDA plates. The bar chart shows colony diameter after incubation for 5 d. (**B**) Pathogenicity assays on apple fruit. The bar charts show lesion diameters on apple fruits at 3 and 5 d post-inoculation. (**C**) Appressorium formation on polyester surfaces at 12 h post-inoculation. Scale bars = 20 µm. The bar chart shows the appressorium formation percentages. (**D**) Turgor pressure assessment of appressoria in the presence of 50% PEG8000. Scale bars = 20 µm. The bar chart shows the percentage of collapsed appressoria. Bars represent the mean ± standard deviation from three independent replicates. Different letters above bars indicate statistically significant differences (*P* < 0.05).

To investigate the significance of these sites, we constructed site-specific mutants of CgCOX6B by substituting each cysteine with serine, resulting in the mutants CgCOX6B^C42S^, CgCOX6B^C62S^, and CgCOX6B^C73S^ (Fig. S7). We first assessed the colony growth of these mutants. The results showed that all three site-specific mutants exhibited significantly reduced colony growth compared to the WT, with outcomes similar to the Δ*CgCOX6B* mutant (Fig. 8A). Next, we performed pathogenicity assays on apple fruit to evaluate the impact of these cysteine mutations on fungal pathogenicity. The Δ*CgCOX6B* mutant and all three site-specific mutants displayed significantly smaller lesions compared to the WT strain (Fig. 8B). We then investigated the ability of these mutants to form appressoria on hydrophobic surfaces. After 12 hours of incubation, the appressorium formation rates of the mutants were similar to that of the WT (Fig. 8C). However, the turgor pressure was significantly impaired in all three mutants, as evidenced by a higher frequency of collapsed appressoria under osmotic stress (Fig. 8D). These results indicate that the cysteine residues Cys42, Cys62, and Cys73 are critical for the full functionality of CgCOX6B.

### CgCOX6B plays a critical role in resistance to Iprodione

**Fig 9:**
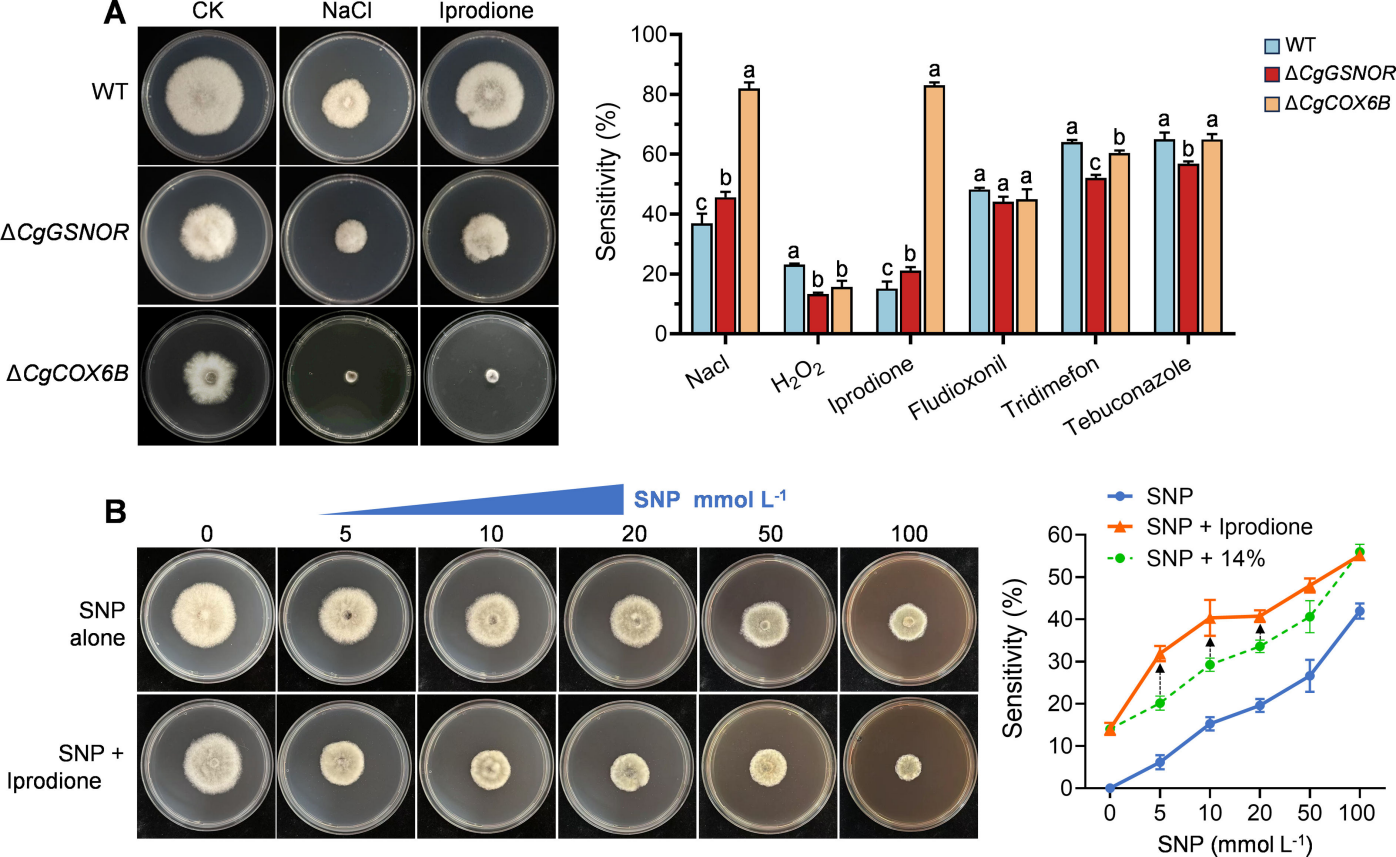
Role of CgCOX6B in stress resistance and synergistic effects of SNP and Iprodione. (**A**) Colony growth sensitivity of WT, Δ*CgGSNOR*, and Δ*CgCOX6B* strains under chemical stresses. The bar chart shows the sensitivity percentages for each strain. Different letters indicate statistically significant differences (*P* < 0.05). (**B**) Dose-dependent effects of sodium nitroprusside (SNP) on *C. gloeosporioides* colony growth when applied alone or in combination with Iprodione. The line chart illustrates the sensitivity to SNP alone or SNP in combination with Iprodione. The green line serves as a reference, representing the additive effect of SNP alone plus 14% inhibition by Iprodione. The black arrows highlight the observed synergistic effects between SNP and Iprodione. Data are presented as mean ± standard deviation from three independent replicates.

The stress resistance of the Δ*CgCOX6B* mutant, particularly its resistance to fungicides, was analyzed under various chemical treatments (Fig. 9A). The results indicated no significant differences in sensitivity to H₂O₂, Triadimefon, Fludioxonil, or Tebuconazole compared to the WT. However, the Δ*CgCOX6B* mutant exhibited markedly increased sensitivity to NaCl and Iprodione. Under these treatments, the Δ*CgCOX6B* mutant showed severely inhibited growth, with almost no detectable colony development (Fig. 9A). In addition, the Δ*CgGSNOR* mutant also displayed increased sensitivity to NaCl and Iprodione, although its growth inhibition was less severe than that observed for the Δ*CgCOX6B* mutant. These findings suggest that CgCOX6B plays a critical role in conferring resistance to NaCl and Iprodione in *C. gloeosporioides*.

Given the observed role of CgCOX6B in stress tolerance, we next investigated whether elevated NO levels could enhance the antifungal activity of Iprodione against *C. gloeosporioides*. SNP was used in combination with Iprodione for treatment. When applied alone, SNP exhibited an inhibitory effect on the colony growth of *C. gloeosporioides* in a dose-dependent manner, with an EC_50_ value of approximately 100 mM, which is impractically high for application purposes. At concentrations of 5 and 10 mmol L^−1^, SNP alone inhibited growth by approximately 6% and 15%, respectively. Similarly, when applied alone, 10 mg L^−1^ Iprodione resulted in an inhibition rate of approximately 14% on colony growth. Interestingly, when SNP and Iprodione were applied simultaneously, SNP at 5 and 10 mmol L^−1^ significantly enhanced the antifungal activity of Iprodione, increasing its inhibition rate to 32% and 40%, representing synergistic increases of 12% and 11%, respectively (Fig. 9B). However, as SNP concentrations were further increased, this synergistic effect gradually diminished. These results strongly suggest that SNP augments Iprodione’s antifungal efficacy by interfering with fungal stress tolerance mechanisms, particularly those mediated by CgCOX6B.

## DISCUSSION

Cysteines are crucial in redox signaling, acting as molecular switches that translate oxidant signals into specific biological responses. The chemical microenvironment surrounding cysteine residues makes them highly reactive toward ROS, NO, and NO-derived reactive nitrogen species (RNS). Among the various reversible post-translational modifications of cysteine, S-nitrosylation stands out as a key regulatory mechanism (41, 42). This modification involves the covalent addition of a NO moiety to the thiol group of cysteine, resulting in the formation of an S-nitrosothiol (SNO). Once formed, S-nitrosothiols can propagate NO signaling through trans-nitrosylation, a thiol/nitrosothiol exchange process that allows NO-mediated signals to extend beyond their site of origin. Low-molecular-weight S-nitrosothiols, such as S-nitrosoglutathione (GSNO), function as effective vectors for transmitting S-nitrosylation signals from their production sites to targeted proteins. To prevent excessive GSNO accumulation and maintain NO homeostasis, cells rely on the enzymatic activity of GSNOR, which degrades GSNO and helps regulate intracellular NO levels (19–21). In *C. gloeosporioides*, the deletion of *CgGSNOR* led to elevated NO levels and increased global protein S-nitrosylation (Fig. 3). This underscores the critical role of *CgGSNOR* in maintaining nitrosative homeostasis and preventing dysregulated NO signaling. Elevated NO levels and heightened S-nitrosylation have been shown to disrupt cellular signaling networks, impair enzymatic activity, and interfere with critical biological processes. These effects were clearly observed in the phenotypic defects in the Δ*CgGSNOR* mutant, including impaired growth, conidiation, appressorium formation, and pathogenicity (Fig. 1 and 2). Further supporting this, we found that exogenous NO negatively impacted appressorium development and turgor pressure in WT strains, and that NO scavenging partially rescued the Δ*CgGSNOR* phenotype (Fig. 4). Moreover, NO exposure increased protein S-nitrosylation levels, particularly in the Δ*CgGSNOR* mutant (Fig. 4C), reinforcing the link between CgGSNOR, NO signaling, and redox regulation.

Mitochondria serve as central hubs for cellular energy production, ROS generation, and stress signaling, making them indispensable for maintaining normal cellular functions and adapting to external stresses. In fungi, these organelles are also critical for pathogenicity, where their capacity to produce ATP and regulate ROS levels directly influences fungal survival and virulence during host invasion (29, 30, 32, 33). Our results demonstrate that the knockout of *CgGSNOR* profoundly disrupts mitochondrial function in *C. gloeosporioides*. The Δ*CgGSNOR* mutant exhibited a substantial reduction in ATP production coupled with altered ROS levels, both of which are hallmarks of mitochondrial dysfunction (Fig. 4). This observation aligns with prior research that disruptions in mitochondrial activity compromise fungal growth and pathogenicity (43). Consistent with these physiological effects, proteomic analysis of mitochondrial S-nitrosylated proteins revealed that key components of the ETC were elevated in S-nitrosylated modification in the Δ*CgGSNOR* mutant (Fig. 5). This suggests a mechanism by which NO accumulation impairs mitochondrial function, contributing to bioenergetic collapse and virulence attenuation.

Previous studies in animal systems have demonstrated that S-nitrosylation of ETC components and proteins involved in mitochondrial fatty acid metabolism can profoundly influence mitochondrial respiration and redox balance, highlighting its regulatory role across various pathophysiological conditions (38, 44). Consistent with these findings, our study revealed that the knockout of *CgGSNOR* led to increased S-nitrosylation of mitochondrial proteins, with numerous ETC components identified as major targets of this modification through proteomic analysis (Fig. 5). Among these targets, COX6B, a smaller subunit of cytochrome c oxidase (COX), emerged as a critical regulator in the biological processes of *C. gloeosporioides*. COX is a central component of the ETC, facilitating electron transfer and contributing to mitochondrial ATP production (45). Within the COX complex, COX6B is crucial for maintaining the stability and functional integrity of the entire ETC assembly (46, 47). In human cells, COX6B dysfunction has been implicated in several diseases, including neurodegenerative disorders and cardiac conditions, where mitochondrial impairment is a hallmark (48, 49). Our findings underscore the vital role of COX6B in fungal biology, as functional validation revealed its involvement in fungal growth, stress adaptation, and pathogenicity. The Δ*CgCOX6B* mutant exhibited severely impaired colony growth, diminished lesion formation, and defective host penetration ability, indicating the importance of COX6B in regulating fungal pathogenicity (Fig. 6). In addition, the Δ*CgCOX6B* mutant exhibited a reduction in mitochondrial ROS levels and ATP content compared to WT, similar to that of Δ*CgGSNOR* (Fig. 6E), indicating that CgCOX6B plays a downstream role in sustaining mitochondrial function under nitrosative stress. To assess whether CgGSNOR directly interacts with CgCOX6B, we performed a yeast two-hybrid (Y2H) assay, which showed no interaction between the two proteins (Fig. S8), suggesting that CgGSNOR modulates CgCOX6B activity indirectly through the regulation of intracellular S-nitrosothiol levels. Moreover, the heightened sensitivity of the Δ*CgCOX6B* mutant to NaCl and Iprodione further suggests that COX6B is required for stress tolerance and fungicide resistance (Fig. 9).

Structural modeling of CgCOX6B provided mechanistic insight into how S-nitrosylation may impair its function. We identified two disulfide bridges, Cys42–Cys62 and Cys32–Cys73, that are likely crucial for protein stability. S-nitrosylation of these cysteine residues led to decreased solvent-accessible surface area and increased structural deviation, particularly at Cys73, as revealed by SASA and RMSD analyses (Fig. 7). These changes suggest that S-nitrosylation disrupts disulfide bond formation and compromises the structural integrity of CgCOX6B, which could account for its loss of function. Our site-directed mutagenesis experiments proved the results of structural modeling, in which the three cysteine residues, Cys42, Cys62, and Cys73, are essential for COX6B function. Mutations at these sites resulted in phenotypes similar to those of the Δ*CgCOX6B* mutant, including reduced colony growth, impaired pathogenicity, and defective turgor pressure maintenance (Fig. 7). COX6B’s role in ATP production further suggests that its S-nitrosylation in the Δ*CgGSNOR* mutant contributes to the reduced ATP levels observed in our study (Fig. 4). Given the central role of ATP in powering cellular processes, this reduction in energy availability likely impairs the fungus’s ability to adapt to environmental challenges and establish successful infections (50–52). Moreover, the disruption in mitochondrial energy metabolism may affect phosphorylation signaling pathways, which are critically dependent on ATP availability (53, 54).

Phosphorylation serves as a fundamental regulatory mechanism for cellular functions, including stress adaptation, growth, and pathogenicity, by modulating the activity of key proteins. Iprodione, a dicarboximide fungicide, is widely employed to control a spectrum of fungal diseases, including postharvest pathogens such as *C. gloeosporioides* (55). Its primary antifungal mechanism centers on the inhibition of protein kinases, thereby disrupting critical signal transduction pathways essential for fungal growth, stress adaptation, and survival. This disruption ultimately culminates in cell death (56). Our findings highlight the critical role of COX6B in conferring resistance to Iprodione (Fig. 8). The significantly reduced ATP levels observed in the Δ*CgGSNOR* mutant (Fig. 4) suggest that the lack of sufficient energy impairs protein kinase activity, leading to diminished phosphorylation signaling. This might further disrupt the pathways necessary for detoxification and stress management, leading to compromises in the fungus’s resistance to Iprodione. This is further supported by our experiments using SNP, which enhanced the antifungal activity of Iprodione against *C. gloeosporioides* (Fig. 8). These findings suggest that targeting the NO signaling pathway and its downstream effectors, including COX6B, offers a promising avenue for enhancing the efficacy of existing fungicides. In addition, this approach may suggest the development of novel antifungal strategies aimed at overcoming resistance and improving crop protection.

The phenotypic differences between the Δ*CgGSNOR* and Δ*CgCOX6B* mutants provide valuable insights into the distinct but interconnected roles of NO signaling and mitochondrial function in fungal development and pathogenicity. While both mutants exhibit impaired growth and reduced pathogenicity, their effects on appressorium formation are notably distinct. In the Δ*CgGSNOR* mutant, appressorium formation is significantly diminished, characterized by abnormal morphology and poorly developed structures (Fig. 2). By contrast, the Δ*CgCOX6B* mutant forms appressoria in numbers comparable to the wild type. However, these appressoria fail to maintain turgor pressure, as evidenced by their increased collapse under osmotic stress (Fig. 6). These findings reveal that while both genes are crucial for fungal pathogenicity, their contributions likely operate through distinct molecular mechanisms. CgGSNOR appears to influence upstream processes critical for appressorium initiation and development, potentially via NO-mediated transcriptional regulation of key genes (28), whereas CgCOX6B functions downstream by ensuring the structural integrity and functional viability of infection structures. This divergence suggests that NO signaling operates at multiple regulatory levels. CgGSNOR likely governs upstream differentiation through NO-mediated signaling, while CgCOX6B plays a downstream role by sustaining mitochondrial energy production and redox balance. The failure of Δ*CgCOX6B* appressoria to maintain turgor pressure correlates with their reduced ATP and ROS levels (Fig. 6E), indicating that energy depletion disrupts osmoregulation and impairs the mechanical force required for host penetration. Beyond direct post-translational modifications such as S-nitrosylation, NO likely exerts significant effects on transcriptional and epigenetic regulation. Previous studies have reported that NO modulates gene expression by influencing transcription factors and epigenetic regulators. For instance, NO may regulate transcription factors such as those from the CREB or WRKY families in animals and plants (10, 57), along with epigenetic components, including histone acetyltransferases and demethylases (58, 59). Recent studies have also revealed broader roles of NO signaling in fungal biology beyond post-translational regulation. In *S. cerevisiae*, NO has been shown to modulate stress-responsive gene networks through S-nitrosylation of enzymes such as transcriptional factors (60). In plant pathogens such as *M. oryzae*, NO accumulation interferes with turgor generation and host penetration, in part by disrupting cytoskeletal organization and redox signaling (28). By contrast, CgCOX6B is likely directly modulated at the protein level through S-nitrosylation, which impacts its enzymatic activity and structural stability, highlighting a different regulatory dimension. This could explain the broader developmental and pathogenic defects observed in the Δ*CgGSNOR* mutant. Future studies should aim to unravel the broader regulatory roles of GSNOR and NO signaling, particularly at the transcriptional and epigenetic levels in fungi. Such insights could pave the way for novel strategies to target key regulatory pathways and mitigate fungal infections.

**Fig 10:**
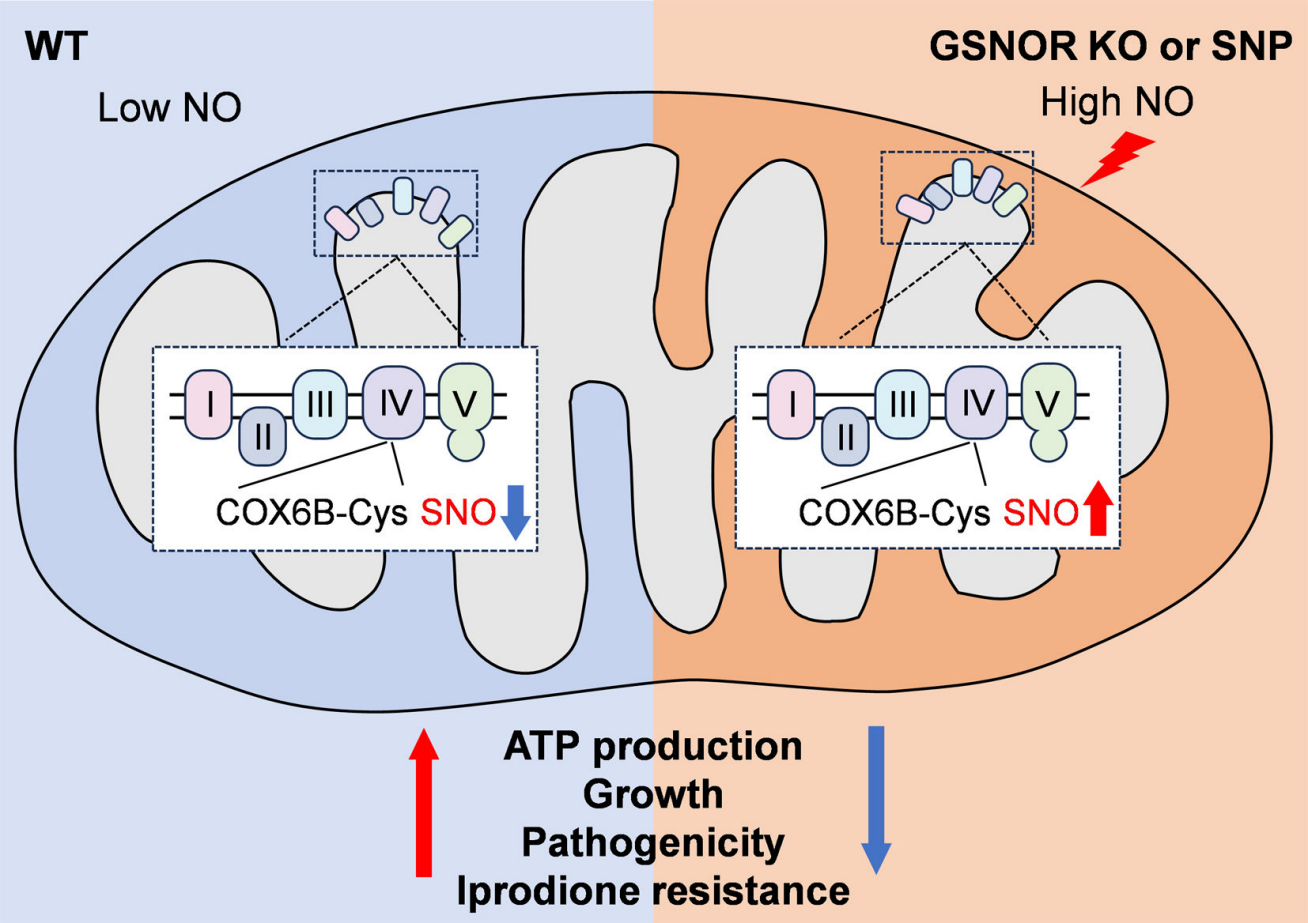
CgGSNOR dynamically regulates CgCOX6B S-nitrosylation to maintain mitochondrial homeostasis, growth, pathogenicity, and Iprodione resistance in *C. gloeosporioides*.

Overall, this study highlights the crucial role of CgGSNOR in regulating NO signaling and its downstream effects, including S-nitrosylation, mitochondrial function, and fungal pathogenicity in *C. gloeosporioides*. Our findings emphasize the significance of cysteine-based redox signaling in fungal physiology, particularly the role of S-nitrosylation in modulating the function of mitochondrial proteins such as CgCOX6B (Fig. 10). Future research should focus on unraveling the precise mechanisms by which S-nitrosylation regulates cellular functions at both the transcriptional and protein levels, which may provide new insights into fungal pathogenesis and disease management for agricultural applications.

## MATERIALS AND METHODS

### Fungal strains and plant materials

The anthracnose causal pathogen *C. gloeosporioides* (BioSample: SAMN17266943) was maintained on PDA medium at 28°C. The colony growth, conidiation, conidial germination, and pathogenicity assays were performed as described previously (61). For the pathogenicity assay, rubber tree leaves (*Hevea brasiliensis*) and apple fruit (*Malus domestica* Borkh. cv. Red Fuji) were selected for inoculation (62).

### Construction of mutant strain

Gene knockouts were generated using the Split-Marker strategy as described previously (61). Briefly, the upstream and downstream flanking fragments of the target gene were amplified and ligated with a truncated acetolactate synthase gene cassette (*SUR*). The recombinant fragments were then used for subsequent protoplast transformation (Fig. S1A). For gene complementation, the gene sequences, along with their native promoters, were cloned into a plasmid containing the terminator from the tryptophan synthase gene of *Aspergillus nidulans* (*TtrpC*) and the hygromycin phosphotransferase gene (*HPT*) (Fig. S1B). The resulting plasmids were linearized before protoplast transformation. To create a TOM20-GFP-tagged strain, the *CgTOM20* gene sequence, including its downstream flanking regions, was amplified and cloned into a plasmid containing the GFP sequence, TtrpC, and HPT (Fig. S1C). This plasmid was then linearized and introduced into protoplasts to allow homologous recombination at the *CgTOM20* loci. For the generation of site-specific mutants, the mutated sequence of *CgCOX6B* was ligated with its native promoter, *TtrpC*, and *HPT*, and subsequently transformed into the *CgCOX6B* knockout mutant protoplasts (Fig. S1D).

Gene knockouts were confirmed through two independent rounds of PCR to verify the correct integration of the recombinant fragments. Further validation of gene deletion was performed using PCR. The complementation strain was confirmed by PCR analysis to detect the presence of the target gene sequence. The site-specific mutant *CgCOX6B* strains were validated by PCR and sequencing to confirm the presence and accuracy of the mutated *CgCOX6B* sequence. Primers used are listed in Table S3.

### Pathogenicity assay

The pathogenicity assays were performed as described previously (62). In brief, “light green” leaves from rubber trees were either kept intact or wounded using a sterile needle prior to inoculation with 5 µL droplets of conidial suspensions. After incubation for 3 d, disease symptoms and lesion diameters were recorded. Each treatment was performed with three replicates, each consisting of 10 leaves, and the experiment was repeated three times. For pathogenicity assays on apple fruit, the fruit was wounded at the equator using a sterile nail, creating a 3 mm deep and 3 mm wide wound. The wounded area was air-dried for 2 hours, after which 5 µL droplets of conidial suspension were applied to each wound. After incubation for 3 and 5 d, disease symptoms and lesion diameters were measured. Each treatment was performed in triplicate, with 10 fruits per replicate, and the experiment was repeated three times.

### Appressorium formation, invasive hyphae formation, and appressorium collapse assays

Microscopic assays for appressorium formation, invasive hyphae development, and appressorium collapse were performed as described previously (61). Briefly, droplets of conidial suspension were incubated on polyester film or onion epidermis to induce the formation of appressoria and invasive hyphae. The formation processes were observed under a Leica DM2000 microscope. For the collapse assay, PEG8000 solutions at concentrations of 40% and 50% (wt/vol) were added to the conidial suspension. Each treatment included three replicates, with each replicate consisting of 50 conidia, and the experiment was repeated twice to ensure reproducibility.

### Fluorescence microscopy

Fluorescent dyes, DAF-FM DA and DHE, were purchased from Merck (Germany), and MitoTracker Red FM was purchased from Thermo Fisher Scientific (USA). For cell staining, droplets of conidial suspension were incubated in YCS medium (1% yeast extract, 1% casein, and 2% sucrose) on a glass slide. After 3–4 hours of conidial germination, the conidia were stained with the fluorescent dyes according to the manufacturer’s instructions. Following staining, the conidia were visualized using either a Leica DM6000 fluorescent microscope or a Leica TCS SP8 laser scanning confocal microscope. The Tom20-GFP-tagged strain was incubated and observed using the same method. All microscopy images were analyzed, and relative fluorescence intensity was calculated using ImageJ software.

### Mitochondria isolation and western blotting

Mitochondria were isolated using the Cell Mitochondria Isolation Kit (Beyotime, China) following the manufacturer’s protocol. Briefly, conidia were inoculated into liquid complete medium and incubated for 1 day. The mycelium was then collected, washed with phosphate-buffered saline (PBS), and gently ground into a powder in liquid nitrogen. This powder was used for mitochondrial isolation according to the kit instructions. To ensure the purity of the isolated mitochondria, mitochondrial proteins were analyzed by SDS-PAGE and Western blotting using antibodies against cytoplasmic and mitochondrial marker proteins, including GAPDH, TOM20, and COXIV (all purchased from Beyotime, China). The S-nitrosylation levels of total cellular and mitochondrial proteins were analyzed using the Pierce S-Nitrosylation Western Blot Kit (Thermo Fisher Scientific, USA).

### Proteomics detection of S-Nitrosylated mitochondrial proteins

Proteomic analysis of mitochondrial S-nitrosylation was performed by PTM BIO (China) following the protocol described by Murray et al. (63) with some modifications. First, mitochondrial proteins were labeled using the iodoTMTsixplex Label Reagent Set (Thermo Fisher Scientific, USA), according to the manufacturer’s instructions. The labeled proteins were then enriched by vacuum freeze-drying, followed by digestion at 37°C for 4 hours using trypsin. After digestion, the samples were desalted using C18 spin tips to remove impurities and unincorporated reagents. Subsequently, the Cys-TMT-labeled peptides were resuspended in TBS buffer and enriched using anti-TMT antibody resin (Thermo Fisher Scientific, USA) overnight at 4°C. After incubation, unbound peptides were collected, and the resin was washed five times with wash buffer, three times with TBS buffer, and three times with water to remove non-specific binders. The TMT-labeled peptides were then eluted from the resin four times using 50% acetonitrile and 0.4% trifluoroacetic acid (TFA). The eluted peptides were dried under vacuum and stored for subsequent analysis by LC-MS/MS. The resulting MS/MS data were processed using the MaxQuant search engine (version 1.6.15.0). Peptide modification levels with a fold change greater than 1.3 and a *P*-value of less than 0.05 were considered significantly altered.

### Protein structural modeling

The three-dimensional structure of the wild-type CgCOX6B protein and its SNC-modified variants were predicted using AlphaFold Server with default settings. Structural alignments between wild-type and SNC-modified models were performed using PyMOL, and RMSD values were calculated to assess conformational differences. SASA was also computed in PyMOL to evaluate the effects of S-nitrosylation on surface exposure.

### Stress tolerance assay

Stress tolerance assays were conducted by culturing colonies on minimal medium supplemented with 30 mmol L^−1^ H_2_O_2_, 0.7 mol L^−1^ NaCl, 10 mg L^−1^ Iprodione, 20 mg L^−1^ Triadimefon, 0.1 mg L^−1^ fludioxonil, and 0.2 mg L^−1^ Tebuconazole, respectively. The strain cultured without any chemical treatment served as the control (CK). The relative sensitivity of the strains to each stressor was calculated using the following formula: Sensitivity (%) = (Colony area of CK – Colony area of treated sample)/(Colony area of CK) × 100. Chemicals were purchased from Sangon Biotech (China) and Merck (Germany). Each treatment consisted of three replicates, and all experiments were performed in duplicate to ensure reproducibility.

### Statistical analysis

Data were analyzed using either Student’s *t*-test or one-way ANOVA with Tukey’s multiple range test, performed via SPSS 18 or GraphPad Prism 8. Differences were considered statistically significant when *P* < 0.05 and highly significant when *P* < 0.01.

## SUPPLEMENTAL MATERIAL

Supplemental Figures mbio.01269-25-s0001.docx Figures S1 to S8.

Supplemental Tables mbio.01269-25-s0002.xlsx Tables S1 to S3.

ASM does not own the copyrights to Supplemental Material that may be linked to, or accessed through, an article. The authors have granted ASM a non-exclusive, world-wide license to publish the Supplemental Material files. Please contact the corresponding author directly for reuse.

## References

[1] Lamattina L, García-Mata C, Graziano M, Pagnussat G. 2003. Nitric oxide: the versatility of an extensive signal molecule. Annu Rev Plant Biol 54:109–136. doi:10.1146/annurev.arplant.54.031902.134752 14502987

[2] Thomas DD, Ridnour LA, Isenberg JS, Flores-Santana W, Switzer CH, Donzelli S, Hussain P, Vecoli C, Paolocci N, Ambs S, Colton CA, Harris CC, Roberts DD, Wink DA. 2008. The chemical biology of nitric oxide: implications in cellular signaling. Free Radic Biol Med 45:18–31. doi:10.1016/j.freeradbiomed.2008.03.020 18439435 PMC2572721

[3] Radi R. 2018. Oxygen radicals, nitric oxide, and peroxynitrite: redox pathways in molecular medicine. Proc Natl Acad Sci USA 115:5839–5848. doi:10.1073/pnas.1804932115 29802228 PMC6003358

[4] Zorov DB, Juhaszova M, Sollott SJ. 2014. Mitochondrial reactive oxygen species (ROS) and ROS-induced ROS release. Physiol Rev 94:909–950. doi:10.1152/physrev.00026.2013 24987008 PMC4101632

[5] Scheler C, Durner J, Astier J. 2013. Nitric oxide and reactive oxygen species in plant biotic interactions. Curr Opin Plant Biol 16:534–539. doi:10.1016/j.pbi.2013.06.020 23880111

[6] Martínez-Medina A, Pescador L, Terrón-Camero LC, Pozo MJ, Romero-Puertas MC. 2019. Nitric oxide in plant-fungal interactions. J Exp Bot 70:4489–4503. doi:10.1093/jxb/erz289 31197351

[7] Domingos P, Prado AM, Wong A, Gehring C, Feijo JA. 2015. Nitric oxide: a multitasked signaling gas in plants. Mol Plant 8:506–520. doi:10.1016/j.molp.2014.12.010 25680232

[8] Tada Y, Mori T, Shinogi T, Yao N, Takahashi S, Betsuyaku S, Sakamoto M, Park P, Nakayashiki H, Tosa Y, Mayama S. 2004. Nitric oxide and reactive oxygen species do not elicit hypersensitive cell death but induce apoptosis in the adjacent cells during the defense response of oat. Mol Plant Microbe Interact 17:245–253. doi:10.1094/MPMI.2004.17.3.245 15000391

[9] Klessig DF, Durner J, Noad R, Navarre DA, Wendehenne D, Kumar D, Zhou JM, Shah J, Zhang S, Kachroo P, Trifa Y, Pontier D, Lam E, Silva H. 2000. Nitric oxide and salicylic acid signaling in plant defense. Proc Natl Acad Sci USA 97:8849–8855. doi:10.1073/pnas.97.16.8849 10922045 PMC34022

[10] Imran QM, Hussain A, Mun BG, Lee SU, Asaf S, Ali MA, Lee IJ, Yun BW. 2018. Transcriptome wide identification and characterization of NO-responsive WRKY transcription factors in Arabidopsis thaliana L. Environ Exp Bot 148:128–143. doi:10.1016/j.envexpbot.2018.01.010

[11] Lindermayr C, Sell S, Müller B, Leister D, Durner J. 2010. Redox regulation of the NPR1-TGA1 system of Arabidopsis thaliana by nitric oxide. Plant Cell 22:2894–2907. doi:10.1105/tpc.109.066464 20716698 PMC2947166

[12] Jedelská T, Luhová L, Petřivalský M. 2021. Nitric oxide signalling in plant interactions with pathogenic fungi and oomycetes. J Exp Bot 72:848–863. doi:10.1093/jxb/eraa596 33367760

[13] Chaudhari SS, Kim M, Lei S, Razvi F, Alqarzaee AA, Hutfless EH, Powers R, Zimmerman MC, Fey PD, Thomas VC. 2017. Nitrite derived from endogenous bacterial nitric oxide synthase activity promotes aerobic respiration. MBio 8:e00887-17. doi:10.1128/mBio.00887-17 28765220 PMC5539425

[14] Zhao Y, Lim J, Xu J, Yu JH, Zheng W. 2020. Nitric oxide as a developmental and metabolic signal in filamentous fungi. Mol Microbiol 113:872–882. doi:10.1111/mmi.14465 31968137

[15] Bando H, Lee Y, Sakaguchi N, Pradipta A, Ma JS, Tanaka S, Cai Y, Liu J, Shen J, Nishikawa Y, Sasai M, Yamamoto M. 2018. Inducible nitric oxide synthase is a key host factor for Toxoplasma GRA15-dependent disruption of the gamma interferon-induced antiparasitic human response. MBio 9:e01738-18. doi:10.1128/mBio.01738-18 30301855 PMC6178625

[16] Foster MW, Hess DT, Stamler JS. 2009. Protein S-nitrosylation in health and disease: a current perspective. Trends Mol Med 15:391–404. doi:10.1016/j.molmed.2009.06.007 19726230 PMC3106339

[17] Fernando V, Zheng X, Walia Y, Sharma V, Letson J, Furuta S. 2019. S-nitrosylation: an emerging paradigm of redox signaling. Antioxidants (Basel) 8:404. doi:10.3390/antiox8090404 31533268 PMC6769533

[18] Fang J, Nakamura T, Cho DH, Gu Z, Lipton SA. 2007. S-nitrosylation of peroxiredoxin 2 promotes oxidative stress-induced neuronal cell death in Parkinson’s disease. Proc Natl Acad Sci USA 104:18742–18747. doi:10.1073/pnas.0705904104 18003920 PMC2141847

[19] Leterrier M, Chaki M, Airaki M, Valderrama R, Palma JM, Barroso JB, Corpas FJ. 2011. Function of S-nitrosoglutathione reductase (GSNOR) in plant development and under biotic/abiotic stress. Plant Signal Behav 6:789–793. doi:10.4161/psb.6.6.15161 21543898 PMC3218474

[20] Li B, Sun C, Lin X, Busch W. 2021. The emerging role of GSNOR in oxidative stress regulation. Trends Plant Sci 26:156–168. doi:10.1016/j.tplants.2020.09.004 33004257

[21] Barnett SD, Buxton ILO. 2017. The role of S-nitrosoglutathione reductase (GSNOR) in human disease and therapy. Crit Rev Biochem Mol Biol 52:340–354. doi:10.1080/10409238.2017.1304353 28393572 PMC5597050

[22] Yun BW, Feechan A, Yin M, Saidi NBB, Le Bihan T, Yu M, Moore JW, Kang JG, Kwon E, Spoel SH, Pallas JA, Loake GJ. 2011. S-nitrosylation of NADPH oxidase regulates cell death in plant immunity. Nature 478:264–268. doi:10.1038/nature10427 21964330

[23] Tada Y, Spoel SH, Pajerowska-Mukhtar K, Mou Z, Song J, Wang C, Zuo J, Dong X. 2008. Plant immunity requires conformational changes [corrected] of NPR1 via S-nitrosylation and thioredoxins. Science 321:952–956. doi:10.1126/science.1156970 18635760 PMC3833675

[24] Cheng T, Chen J, Ef AA, Wang P, Wang G, Hu X, Shi J. 2015. Quantitative proteomics analysis reveals that S-nitrosoglutathione reductase (GSNOR) and nitric oxide signaling enhance poplar defense against chilling stress. Planta 242:1361–1390. doi:10.1007/s00425-015-2374-5 26232921

[25] Zhou S, Jia L, Chu H, Wu D, Peng X, Liu X, Zhang J, Zhao J, Chen K, Zhao L. 2016. Arabidopsis CaM1 and CaM4 promote nitric oxide production and salt resistance by inhibiting S-nitrosoglutathione reductase via direct binding. PLoS Genet 12:e1006255. doi:10.1371/journal.pgen.1006255 27684709 PMC5042403

[26] Liu R, Zhu T, Chen X, Wang Z, Yang Z, Ren A, Shi L, Yu H, Zhao M. 2022. GSNOR regulates ganoderic acid content in Ganoderma lucidum under heat stress through S-nitrosylation of catalase. Commun Biol 5:32. doi:10.1038/s42003-021-02988-0 35017648 PMC8752759

[27] Astuti RI, Watanabe D, Takagi H. 2016. Nitric oxide signaling and its role in oxidative stress response in Schizosaccharomyces pombe. Nitric Oxide 52:29–40. doi:10.1016/j.niox.2015.11.001 26645666

[28] Hu H, He W, Qu Z, Dong X, Ren Z, Qin M, Liu H, Zheng L, Huang J, Chen XL. 2024. De-nitrosylation coordinates appressorium function for infection of the rice blast fungus. Adv Sci (Weinh) 11:e2403894. doi:10.1002/advs.202403894 38704696 PMC11234416

[29] Shingu-Vazquez M, Traven A. 2011. Mitochondria and fungal pathogenesis: drug tolerance, virulence, and potential for antifungal therapy. Eukaryot Cell 10:1376–1383. doi:10.1128/EC.05184-11 21926328 PMC3209048

[30] Verma S, Shakya VPS, Idnurm A. 2018. Exploring and exploiting the connection between mitochondria and the virulence of human pathogenic fungi. Virulence 9:426–446. doi:10.1080/21505594.2017.1414133 29261004 PMC5955198

[31] Egan MJ, Wang ZY, Jones MA, Smirnoff N, Talbot NJ. 2007. Generation of reactive oxygen species by fungal NADPH oxidases is required for rice blast disease. Proc Natl Acad Sci USA 104:11772–11777. doi:10.1073/pnas.0700574104 17600089 PMC1913907

[32] Chang AL, Doering TL. 2018. Maintenance of mitochondrial morphology in Cryptococcus neoformans is critical for stress resistance and virulence. MBio 9:e01375-18. doi:10.1128/mBio.01375-18 30401774 PMC6222134

[33] Horianopoulos LC, Hu G, Caza M, Schmitt K, Overby P, Johnson JD, Valerius O, Braus GH, Kronstad JW. 2020. The novel J-domain protein Mrj1 is required for mitochondrial respiration and virulence in Cryptococcus neoformans. MBio 11:01127–01120. doi:10.1128/mBio.01127-20 PMC7373193 32518190

[34] An B, Li B, Li H, Zhang Z, Qin G, Tian S. 2016. Aquaporin8 regulates cellular development and reactive oxygen species production, a critical component of virulence in Botrytis cinerea. New Phytol 209:1668–1680. doi:10.1111/nph.13721 26527167

[35] Liu N, Wang W, He C, Luo H, An B, Wang Q. 2022. NADPH oxidases play a role in pathogenicity via the regulation of F-actin organization in Colletotrichum gloeosporioides. Front Cell Infect Microbiol 12:845133. doi:10.3389/fcimb.2022.845133 35782153 PMC9240266

[36] O’Connell RJ, Thon MR, Hacquard S, Amyotte SG, Kleemann J, Torres MF, Damm U, Buiate EA, Epstein L, Alkan N. 2012. Lifestyle transitions in plant pathogenic Colletotrichum fungi deciphered by genome and transcriptome analyses. Nat Genet 44:1060–1065. doi:10.1038/ng.2372 22885923 PMC9754331

[37] Rizza S, Cardaci S, Montagna C, Di Giacomo G, De Zio D, Bordi M, Maiani E, Campello S, Borreca A, Puca AA, Stamler JS, Cecconi F, Filomeni G. 2018. S -nitrosylation drives cell senescence and aging in mammals by controlling mitochondrial dynamics and mitophagy . Proc Natl Acad Sci USA 115. doi:10.1073/pnas.1722452115 PMC5899480 29581312

[38] Montagna C, Cirotti C, Rizza S, Filomeni G. 2020. When S-nitrosylation gets to mitochondria: from signaling to age-related diseases. Antioxid Redox Signal 32:884–905. doi:10.1089/ars.2019.7872 31931592

[39] Clementi E, Brown GC, Feelisch M, Moncada S. 1998. Persistent inhibition of cell respiration by nitric oxide: crucial role of S-nitrosylation of mitochondrial complex I and protective action of glutathione. Proc Natl Acad Sci USA 95:7631–7636. doi:10.1073/pnas.95.13.7631 9636201 PMC22706

[40] Burwell LS, Nadtochiy SM, Tompkins AJ, Young S, Brookes PS. 2006. Direct evidence for S-nitrosation of mitochondrial complex I. Biochem J 394:627–634. doi:10.1042/BJ20051435 16371007 PMC1383712

[41] Bak DW, Weerapana E. 2015. Cysteine-mediated redox signalling in the mitochondria. Mol Biosyst 11:678–697. doi:10.1039/c4mb00571f 25519845

[42] Moldogazieva NT, Mokhosoev IM, Feldman NB, Lutsenko SV. 2018. ROS and RNS signalling: adaptive redox switches through oxidative/nitrosative protein modifications. Free Radic Res 52:507–543. doi:10.1080/10715762.2018.1457217 29589770

[43] Neubauer M, Zhu Z, Penka M, Helmschrott C, Wagener N, Wagener J. 2015. Mitochondrial dynamics in the pathogenic mold Aspergillus fumigatus: therapeutic and evolutionary implications. Mol Microbiol 98:930–945. doi:10.1111/mmi.13167 26272083

[44] Doulias PT, Tenopoulou M, Greene JL, Raju K, Ischiropoulos H. 2013. Nitric oxide regulates mitochondrial fatty acid metabolism through reversible protein S-nitrosylation. Sci Signal 6:rs1. doi:10.1126/scisignal.2003252 23281369 PMC4010156

[45] Wikström M, Krab K, Sharma V. 2018. Oxygen activation and energy conservation by cytochrome c oxidase. Chem Rev 118:2469–2490. doi:10.1021/acs.chemrev.7b00664 29350917 PMC6203177

[46] Shimada S, Shinzawa-Itoh K, Baba J, Aoe S, Shimada A, Yamashita E, Kang J, Tateno M, Yoshikawa S, Tsukihara T. 2017. Complex structure of cytochrome c-cytochrome c oxidase reveals a novel protein-protein interaction mode. EMBO J 36:291–300. doi:10.15252/embj.201695021 27979921 PMC5286356

[47] Čunátová K, Reguera DP, Houštěk J, Mráček T, Pecina P. 2020. Role of cytochrome c oxidase nuclear-encoded subunits in health and disease. Physiol Res 69:947–965. doi:10.33549/physiolres.934446 33129245 PMC8549878

[48] Bi R, Zhang W, Zhang DF, Xu M, Fan Y, Hu Q-X, Jiang H-Y, Tan L, Li T, Fang Y, Zhang C, Yao YG. 2018. Genetic association of the cytochrome c oxidase-related genes with Alzheimer’s disease in Han Chinese. Neuropsychopharmacology 43:2264–2276. doi:10.1038/s41386-018-0144-3 30054583 PMC6135758

[49] Chen YT, Xu XH, Lin L, Tian S, Wu G-F. 2023. Identification of three cuproptosis-specific expressed genes as diagnostic biomarkers and therapeutic targets for atherosclerosis. Int J Med Sci 20:836–848. doi:10.7150/ijms.83009 37324184 PMC10266043

[50] Vestergaard M, Bald D, Ingmer H. 2022. Targeting the ATP synthase in bacterial and fungal pathogens: beyond Mycobacterium tuberculosis. J Glob Antimicrob Resist 29:29–41. doi:10.1016/j.jgar.2022.01.026 35131507

[51] Li SX, Song YJ, Zhang YS, Wu HT, Guo H, Zhu KJ, Li DM, Zhang H. 2017. Mitochondrial complex V α subunit is critical for Candida albicans pathogenicity through modulating multiple virulence properties. Front Microbiol 8:285. doi:10.3389/fmicb.2017.00285 28280492 PMC5322696

[52] Britton TA, Wu C, Chen YW, Franklin D, Chen Y, Camacho MI, Luong TT, Das A, Ton-That H. 2024. The respiratory enzyme complex Rnf is vital for metabolic adaptation and virulence in Fusobacterium nucleatum. MBio 15:e0175123. doi:10.1128/mbio.01751-23 38059640 PMC10790702

[53] Hardie DG, Schaffer BE, Brunet A. 2016. AMPK: an energy-sensing pathway with multiple inputs and outputs. Trends Cell Biol 26:190–201. doi:10.1016/j.tcb.2015.10.013 26616193 PMC5881568

[54] Herrero-de-Dios C, Day AM, Tillmann AT, Kastora SL, Stead D, Salgado PS, Quinn J, Brown AJP. 2018. Redox regulation, rather than stress-induced phosphorylation, of a Hog1 mitogen-activated protein kinase modulates its nitrosative-stress-specific outputs. MBio 9:e02229-17. doi:10.1128/mBio.02229-17 29588408 PMC5874921

[55] Legard DE, Xiao CL, Mertely JC, Chandler CK. 2001. Management of Botrytis fruit rot in annual winter strawberry using captan, thiram, and iprodione. Plant Dis 85:31–39. doi:10.1094/PDIS.2001.85.1.31 30832067

[56] Sang H, Popko JT Jr, Chang T, Jung G. 2017. Molecular mechanisms involved in qualitative and quantitative resistance to the dicarboximide fungicide iprodione in Sclerotinia homoeocarpa field isolates.. Phytopathology 107:198–207. doi:10.1094/PHYTO-05-16-0211-R 27642797

[57] Riccio A, Alvania RS, Lonze BE, Ramanan N, Kim T, Huang Y, Dawson TM, Snyder SH, Ginty DD. 2006. A nitric oxide signaling pathway controls CREB-mediated gene expression in neurons. Mol Cell 21:283–294. doi:10.1016/j.molcel.2005.12.006 16427017

[58] Nott A, Watson PM, Robinson JD, Crepaldi L, Riccio A. 2008. S-Nitrosylation of histone deacetylase 2 induces chromatin remodelling in neurons. Nature 455:411–415. doi:10.1038/nature07238 18754010

[59] Zheng Y, Li Z, Cui X, Yang Z, Bao C, Pan L, Liu X, Chatel-Innocenti G, Vanacker H, Noctor G, Dard A, Reichheld JP, Issakidis-Bourguet E, Zhou DX. 2023. S-Nitrosylation of the histone deacetylase HDA19 stimulates its activity to enhance plant stress tolerance in Arabidopsis. Plant J 114:836–854. doi:10.1111/tpj.16174 36883867

[60] Merhej J, Delaveau T, Guitard J, Palancade B, Hennequin C, Garcia M, Lelandais G, Devaux F. 2015. Yap7 is a transcriptional repressor of nitric oxide oxidase in yeasts, which arose from neofunctionalization after whole genome duplication. Mol Microbiol 96:951–972. doi:10.1111/mmi.12983 25732006

[61] Zhang Y, An B, Wang W, Zhang B, He C, Luo H, Wang Q. 2022. Actin-bundling protein fimbrin regulates pathogenicity via organizing F-actin dynamics during appressorium development in Colletotrichum gloeosporioides. Mol Plant Pathol 23:1472–1486. doi:10.1111/mpp.13242 35791045 PMC9452767

[62] Wen X, Wang Q, Luo H, He C, An B. 2024. A Baeyer-Villiger monooxygenase CgBVMO1 is involved in superoxide anion metabolism, cell wall synthesis, and pathogenicity of Colletotrichum gloeosporioides. Postharvest Biol Technol 210:112786. doi:10.1016/j.postharvbio.2024.112786

[63] Murray CI, Uhrigshardt H, O’Meally RN, Cole RN, Van Eyk JE. 2012. Identification and quantification of S-nitrosylation by cysteine reactive tandem mass tag switch assay. Mol Cell Proteomics 11:M111. doi:10.1074/mcp.M111.013441 PMC3277766 22126794

