# Supplementary material for: GSNOR plays roles in growth, pathogenicity, and stress resistance by modulating mitochondrial protein COX6B S-nitrosylation in Colletotrichum gloeosporioides
Source: mBio. 2025 May 23;16(6):e01269-25. doi: 10.1128/mbio.01269-25 (PMC12153361; doi:10.1128/mbio.01269-25)
Supplement: Supplemental Figures — Figures S1 to S8. [file mbio.01269-25-s0001.docx]

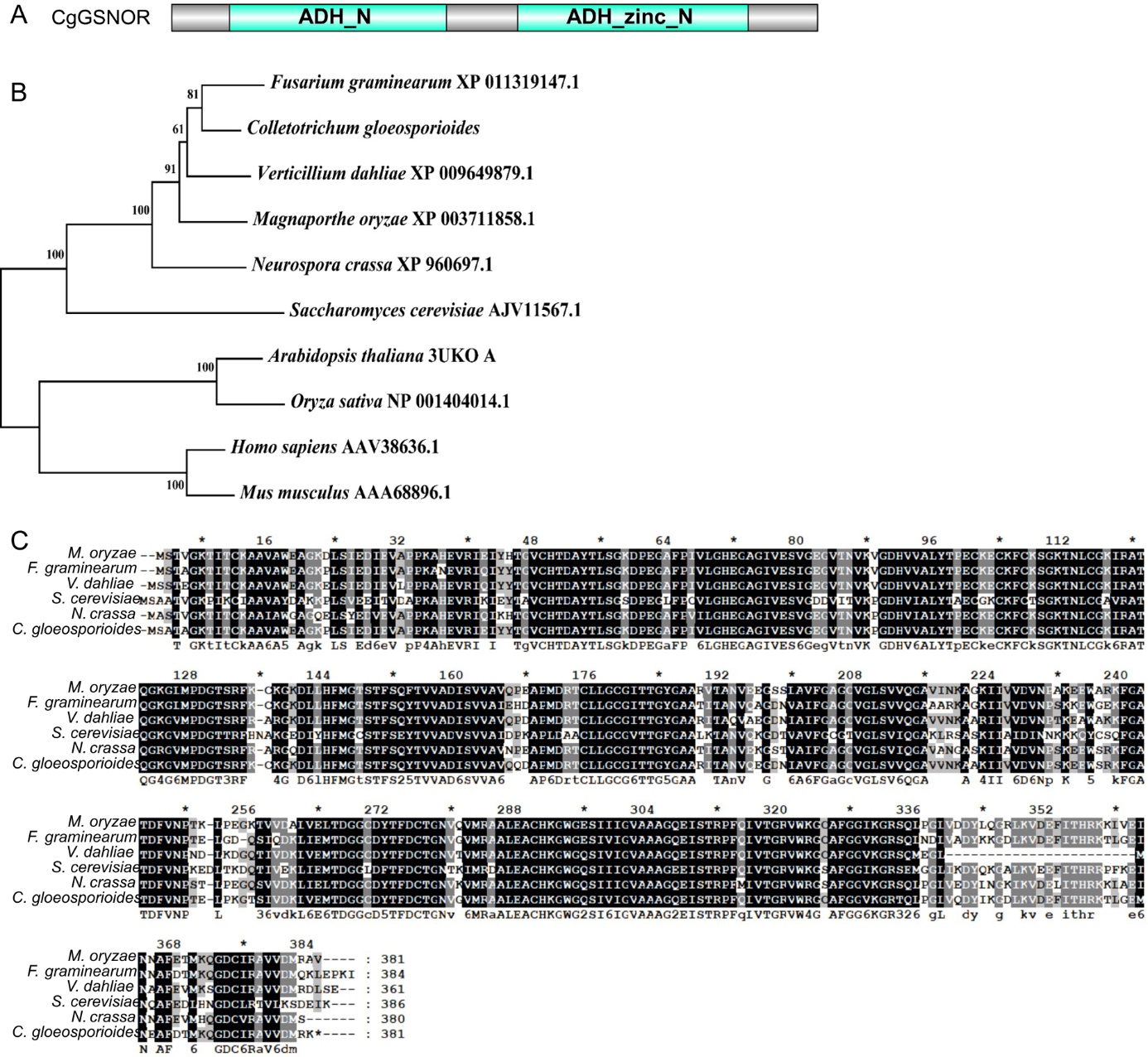


**FIG S1 Structural and evolutionary analysis of CgGSNOR. (A)** Schematic representation of *CgGSNOR* conserved domains, including Alcohol dehydrogenase GroES-like (ADH_N) and Zinc-binding dehydrogenase (ADH_zinc_N) domains. **(B)** Phylogenetic tree of *CgGSNOR* and its homologs from various species, including plant-pathogenic fungi (*Fusarium graminearum*, *Verticillium dahliae*), model fungi (*Neurospora crassa*, *Saccharomyces cerevisiae*), plants (*Arabidopsis thaliana*, *Oryza sativa*), and mammals (*Homo sapiens*, *Mus musculus*). The tree was constructed using the maximum likelihood method, and bootstrap values are shown at the nodes. **(C)** Multiple sequence alignment of *CgGSNOR* with its homologs. Conserved amino acid residues are highlighted in black and gray.


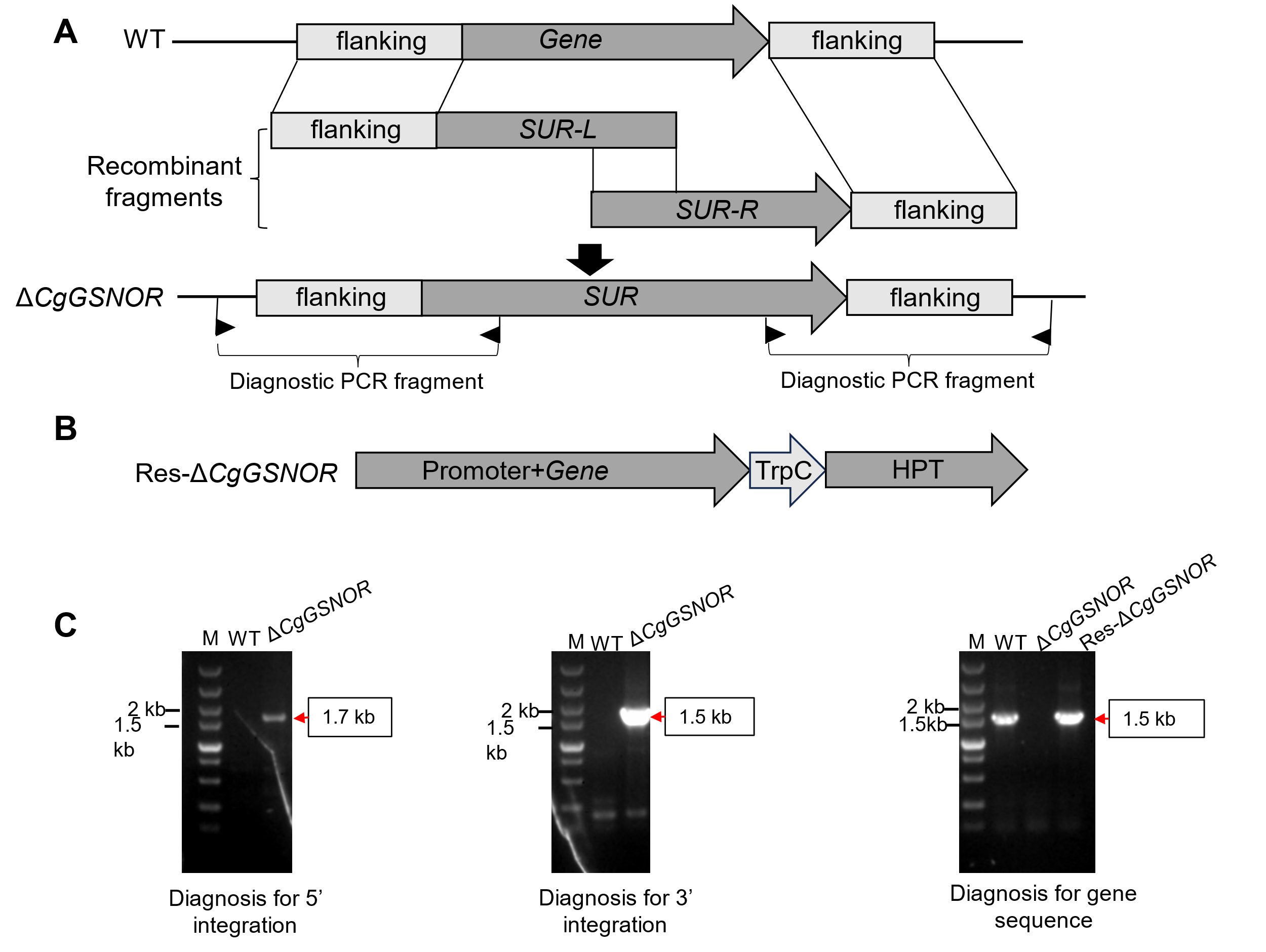


**FIG S2 Construction of the *CgGSNOR* knockout mutant. (A)** Schematic representation of the homologous recombination strategy used for gene knockout. The *Magnaporthe oryzae* acetolactate synthase gene cassette conferring resistance to chlorimuron ethyl was used as a selective marker. PCR primers, represented by black triangles, were designed to detect the integration of recombinant fragments and confirm the deletion of the target gene. WT: wide type; the symbol “Δ” indicates the gene knockout mutants. (**B)** Diagram illustrating the complementation strategy for the *CgGSNOR* knockout mutant. The full-length coding sequence of *CgGSNOR* and its native 1 kb promoter were cloned into a plasmid containing the *Aspergillus nidulans* tryptophan synthase terminator (TtrpC) and the hygromycin phosphotransferase gene (HPT) as a selectable marker. **(C)** PCR analysis confirming the correct integration of recombinant fragments at the target locus and verifying the presence of the gene.


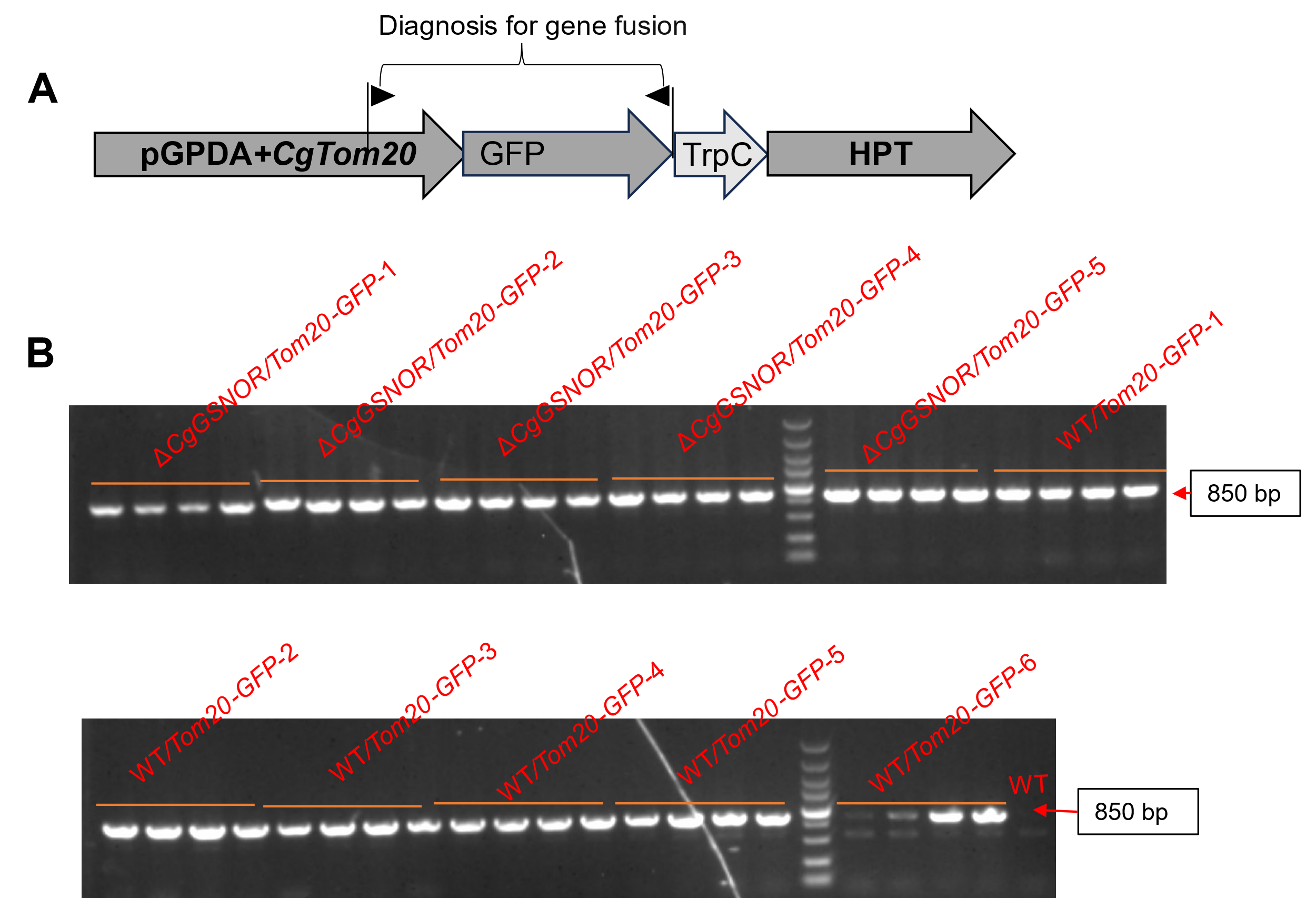


**FIG S3 Construction of the TOM20-GFP fusion expression mutants. (A)** Schematic representation of the construct used for generating the *CgTOM20*-GFP fusion expression mutant. The full-length coding region of *CgTOM20* along with its native 1 kb promoter sequence was cloned into a plasmid containing the GFP coding sequence. The plasmid also included the terminator of *Aspergillus nidulans* tryptophan synthase (TtrpC) and the hygromycin phosphotransferase gene (HPT) as a selectable marker. **(B)** PCR analysis confirming the presence of the *CgTOM20*-GFP fusion sequence.


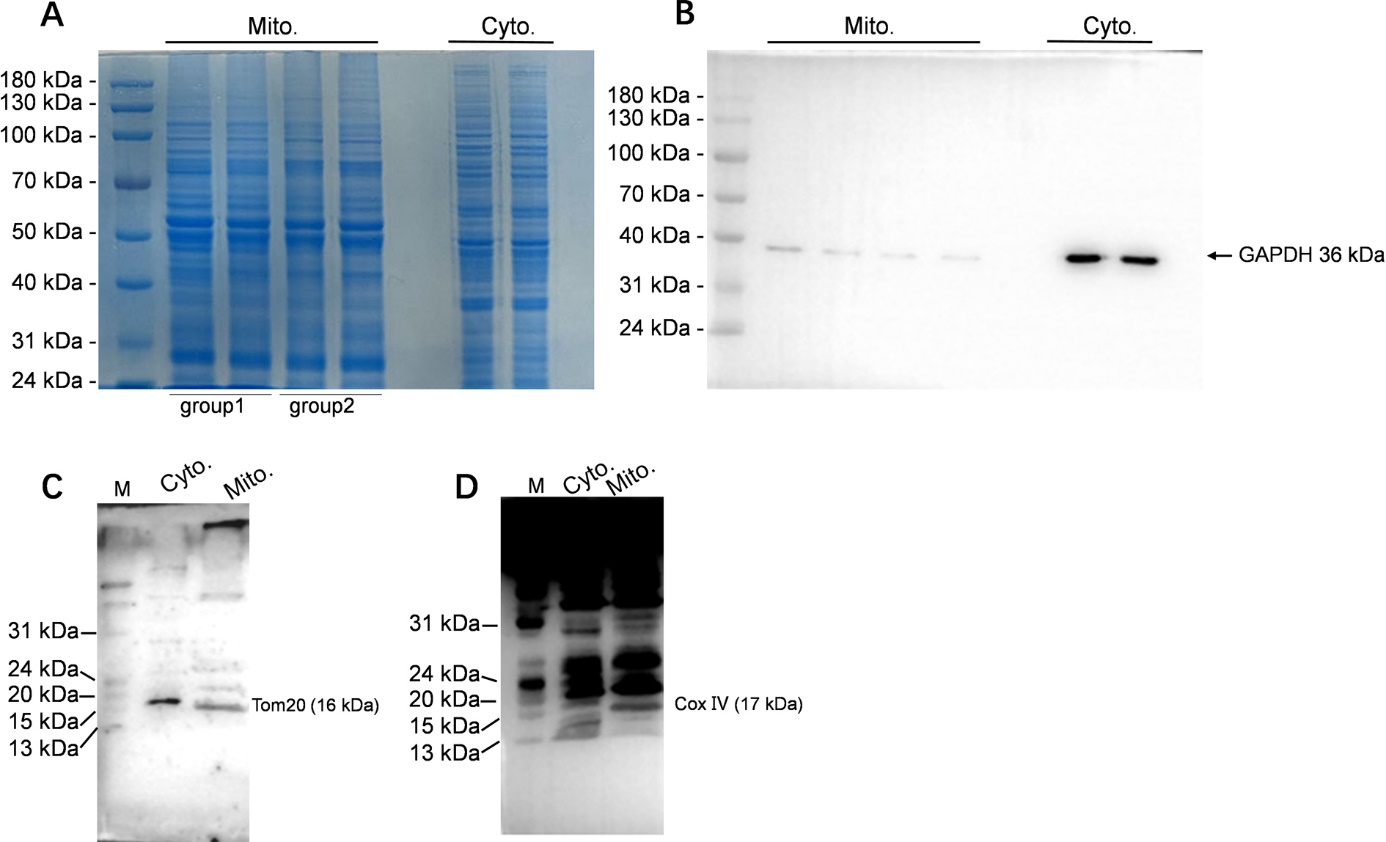


**FIG S4 Quality assessment of isolated mitochondria from *C. gloeosporioides*. (A)** SDS-PAGE analysis of protein samples from mitochondrial (Mito.) and cytoplasmic (Cyto.) fractions. Mitochondria were isolated using two different protocols (group 1 and group 2) following the manufacturer’s guidelines. **(B)** Western blot analysis of GAPDH in mitochondrial and cytoplasmic fractions. **(C)** Western blot analysis of Tom20 in mitochondrial and cytoplasmic fractions. **(D)** Western blot analysis of Cox IV in mitochondrial and cytoplasmic fractions.


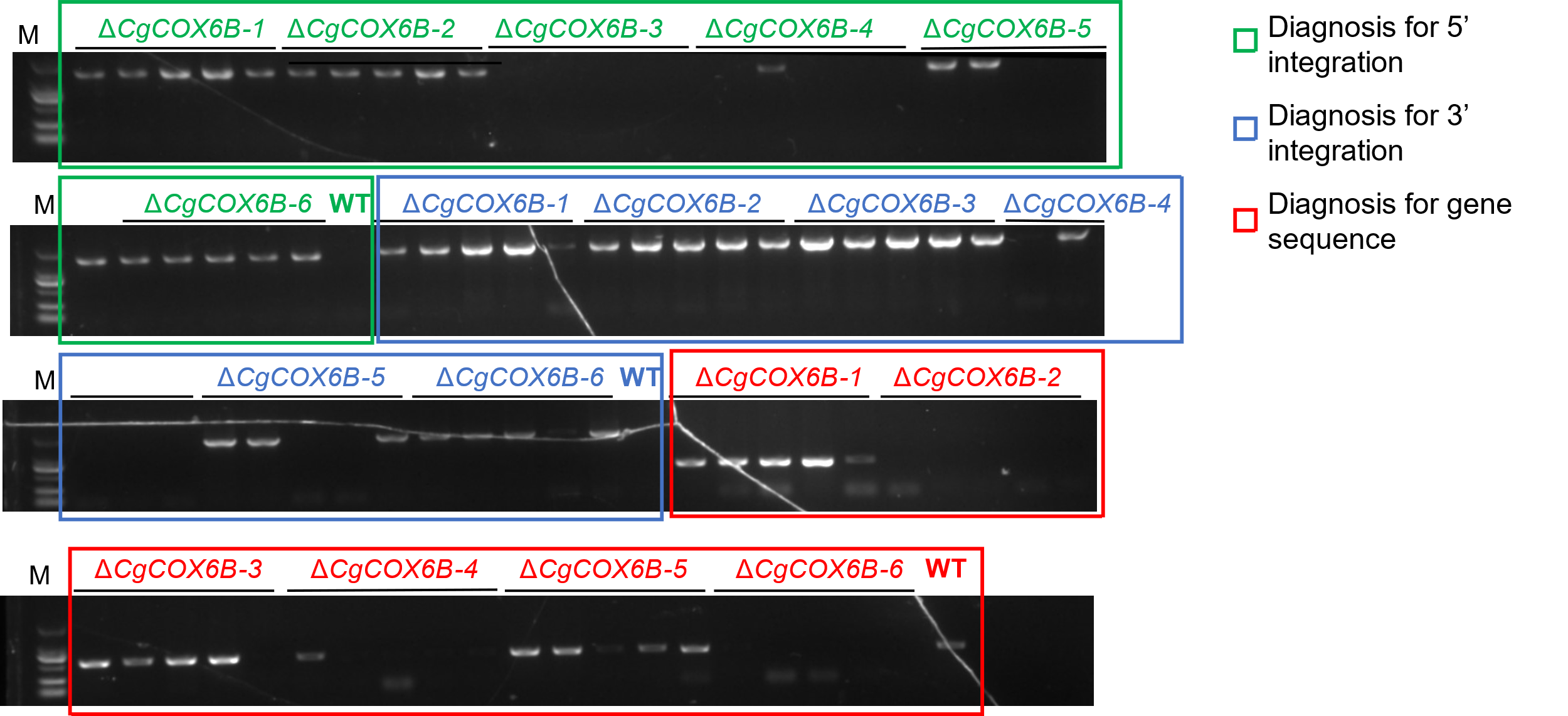


**FIG S5 Diagnosis for the *CgCOX6B* knockout mutants.** The correct integration of the recombinant fragments into the right target locus, and the presence of the desired genes were analyzed.


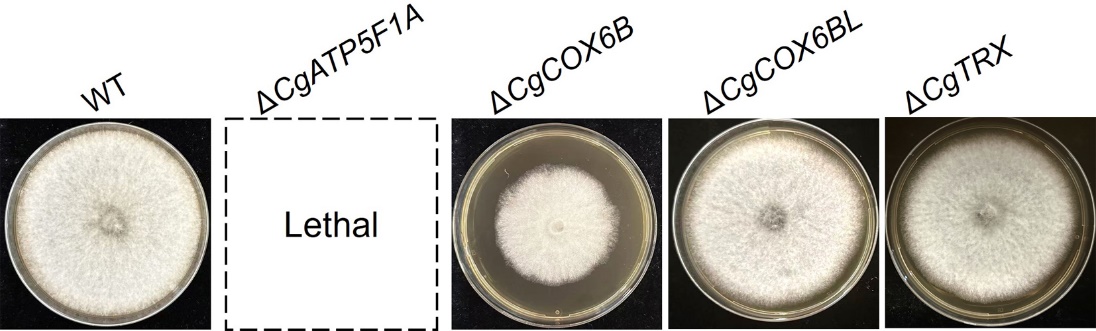


**FIG S6** Colony growth of relative electron transport chain protein mutants.


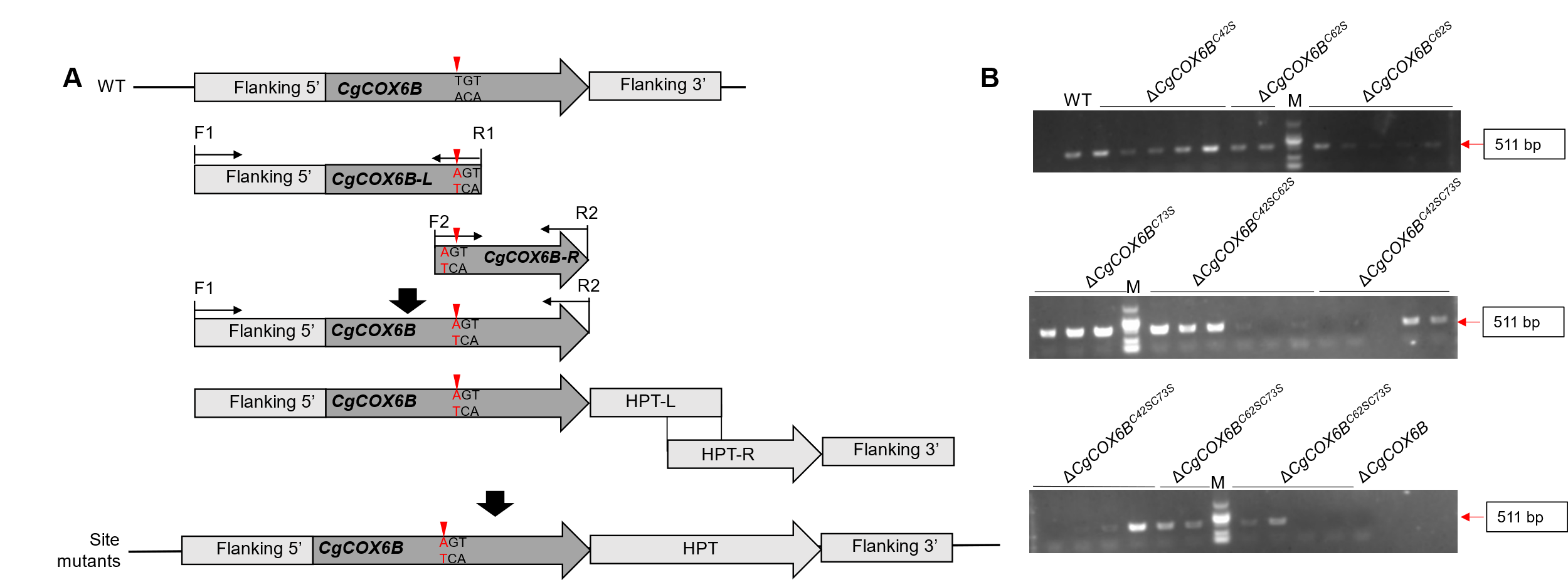


**FIG S7 Construction of the CgCOX6B site specific mutants. (A)** Strategy diagram illustrating the generation of site-specific mutants for CgCOX6B. The mutated *CgCOX6B* sequence and its downstream flanking were ligated with the hygromycin phosphotransferase gene (*HPT*), and used for replacement of the native CgCOX6B sequence through homologous recombination. **(B)** PCR analysis and sequencing confirming the presence of the site-specific mutations.

**
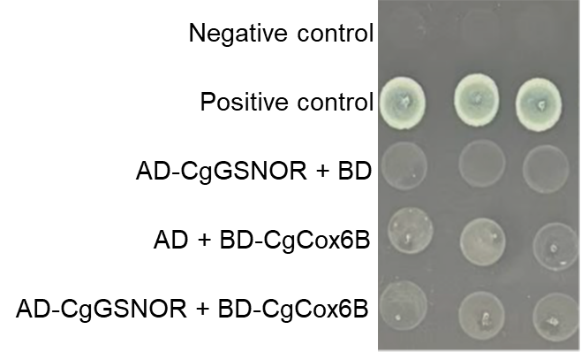
**

**FIG S8** Yeast two-hybrid (Y2H) assay showing that CgGSNOR does not interact with CgCox6B.

Yeast cells co-transformed with the indicated constructs were grown on SD/-Leu/-Trp/-His/-Ade selective medium.
